# Green synthesis of gum Arabic-activated magnetite–nickel nanoparticles for selective removal of Cd(ii) and Cu(ii) from complex aqueous systems

**DOI:** 10.1039/d5ra04152j

**Published:** 2025-09-25

**Authors:** Entsar H. Taha, Adel A. El-Zahhar, Majed M. Alghamdi, Ahmed M. Masoud, Mohamed F. Kamel, Mohamed H. Taha

**Affiliations:** a Department of Plant Protection, Faculty of Agriculture, Ain Shams University, Egypt, Nuclear Materials Authority P. O. Box 530, El Maddi Cairo Egypt tahaentesar214@gmail.com; b Department of Chemistry, Faculty of Science, King Khalid University Abha 9004 Saudi Arabia; c Nuclear Materials Authority P. O. Box 530, El Maddi Cairo Egypt

## Abstract

Despite the growing interest in biofunctionalized magnetic nanocomposites, there is limited research on integrating nickel with gum Arabic (GA) to enhance selectivity and stability for heavy metal removal in complex aqueous systems. A green, biofunctional magnetite–nickel nanocomposite (GA-NiMMPs) was synthesized *via* a modified co-precipitation method using GA as a natural stabilizer and surface modifier. Cadmium and copper were selected as target contaminants because they are among the most hazardous and prevalent heavy metals in industrial effluents, frequently co-existing in electroplating, mining, and metal-finishing wastewaters. GA-NiMMPs exhibited a surface area of 29.32 m^2^ g^−1^, a pore diameter of 1.91 nm, and a zeta potential of −22.5 mV, favoring the adsorption of divalent metal ions and enabling their simultaneous removal. Batch adsorption experiments were conducted using 50 mg per L initial concentrations, varying pH, dosage, and contact time. High removal efficiencies of 93.5% for Cd(ii) and 89.0% for Cu(ii), with maximum capacities of 30.4 and 27.7 mg g^−1^, respectively, were obtained with optimal performance at pH 6.0, 4.0 g per L dosage, and 240 min contact time. Kinetics follow a pseudo-second-order model, while isotherms fit Langmuir and Sips models, indicating monolayer chemisorption as described by the Langmuir model, alongside surface heterogeneity captured by the Sips model. The material showed higher affinity for Cd(ii), achieving 64% selectivity in binary and 58% in multicomponent systems. These results underscore GA-NiMMPs as a promising bio-derived adsorbent for sustainable Cd(ii) and Cu(ii) remediation in complex waters.

## Abstract 2

Nickel-incorporated gum Arabic–magnetite nanocomposites (GA–NiMMPs) were synthesized *via* a green route, showing enhanced selectivity and stability for Cd(ii) and Cu(ii) removal from complex aqueous systems.

## Graphical abstract

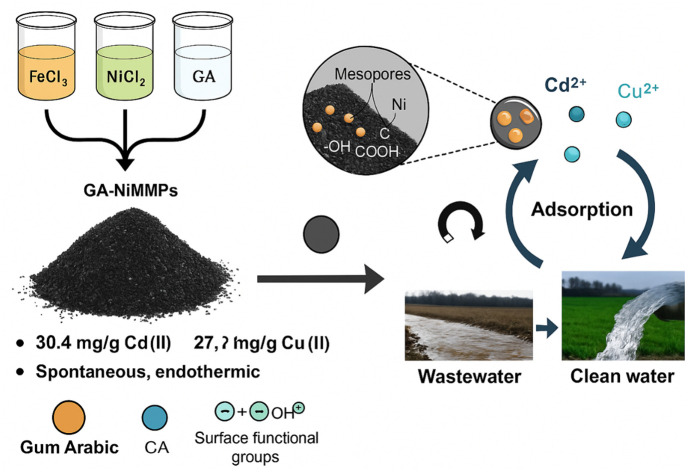

## 1. Introduction

Water contamination by heavy metals poses a persistent and escalating global environmental challenge due to the toxicity, persistence, and bioaccumulative nature of these pollutants in aquatic systems.^1–3^ Among them, cadmium (Cd) and copper (Cu) are particularly hazardous, owing to their extensive industrial applications—including electroplating, battery manufacturing, electronics, and mining—and their adverse health and ecological consequences. Cadmium exposure is linked to renal dysfunction, bone demineralization, and carcinogenic risks, whereas excessive copper levels can cause hepatic and neurological impairments.^3,4^ In aquatic ecosystems, both metals disrupt biodiversity and affect reproductive cycles of marine organisms. Regulatory agencies such as the World Health Organization (WHO) and the United States Environmental Protection Agency (USEPA) have established maximum permissible concentrations of 0.003 mg L^−1^ for Cd and 2 mg L^−1^ for Cu in drinking water.^3–5^ Despite these stringent thresholds, industrial discharges and inadequate treatment technologies frequently lead to exceedances, highlighting the urgent need for more efficient and sustainable remediation strategies.

Traditional heavy metal removal technologies—including chemical precipitation,^6^ ion exchange,^7^ membrane filtration,^8^ and solvent extraction^9^—have been widely applied but often suffer from drawbacks such as high operational costs, energy demand, sludge generation, and limited metal selectivity. For example, chemical precipitation produces hazardous sludge requiring costly post-treatment, membrane systems are prone to fouling and demand frequent maintenance, ion exchange resins are efficient but expensive, and solvent extraction may introduce organic solvent residues that cause secondary pollution.^1–4^ These limitations underscore the need for alternative solutions that are both environmentally benign and economically viable.

As a result, there is a growing demand for cost-effective, environmentally sustainable, and highly efficient materials for heavy metal removal. Adsorption has emerged as a promising approach due to its simplicity, affordability, and ability to remove trace-level contaminants with high precision.^1–4^ Recent research has explored a wide range of adsorbents, including natural sorbents, synthetic polymers, and magnetic nanoparticles. These materials can be grouped by type, such as: (i) carbon-based biochars (*e.g.*, from willow wood and cattle manure,^10^ and Bael fruit shell-derived carbon^2^), (ii) biopolymer-based composites (*e.g.*, chitosan-vermiculite,^11^ Ag-MOF/chitosan sponge^12^), (iii) inorganic frameworks (*e.g.*, zeolites^13^), and (iv) advanced materials like sulfidized nanoscale zero-valent iron (S-nZVI) and sulfur-functionalized activated carbons.^14–16^ While informative, long listings of such sorbents often obscure comparative insight; here we focus on materials that combine high affinity with reusability and recoverability.

The development of advanced adsorbents with high surface area, enhanced binding affinity, and superior selectivity is crucial for optimizing adsorption efficiency. Magnetite-based nanoparticles have gained significant attention due to their unique magnetic properties, ease of recovery, and potential for functionalization.^17–19^ The use of plant-derived polysaccharides—such as gum Arabic (GA)—in the synthesis of these materials is a compelling strategy for biomass valorization. Gum Arabic is a naturally abundant, biodegradable exudate obtained from *Acacia* species, composed primarily of arabinogalactan polysaccharides with ample hydroxyl and carboxyl groups suitable for metal ion binding.^20–22^ It functions as a stabilizing agent, functionalizing ligand, and dispersant, enabling uniform particle distribution and preventing agglomeration.^20–22^ Recent studies have demonstrated the effectiveness of GA-modified sorbents in toxic elements uptake. GA-magnetite nanoparticles (GA/MNPs) efficiently adsorb Pb(ii) (50.5 mg g^−1^),^23^ while GA-functionalized magnetic nanoparticles (GA-MNPs) achieve a 96.3% removal rate of ciprofloxacin (CIP).^22^ GA-poly(vinyl alcohol) (PVA) hydrogels exhibit strong affinities for Pb(ii), Cu(ii), Co(ii), and Cd(ii), making them promising for industrial wastewater treatment.^21^ Additionally, GA-alginate beads, with and without magnetite, show enhanced adsorption of Cu(ii), Cd(ii), and Pb(ii).^24^ Moreover, composites of GA with TiO_2_, Al_2_O_3_, or MgO nanoparticles exhibit remarkable performance in multi-metal adsorption, benefiting from high surface areas and active binding sites.^20^ Nickel incorporation into the magnetite lattice introduces lattice distortions and enhances surface redox properties, thereby increasing the number and diversity of active sites available for metal ion binding. This modification facilitates improved adsorption capacity and selectivity, particularly for divalent ions such as Cd(ii) and Cu(ii).^1,25–28^ Recent studies have demonstrated that nickel-based ferrites and Ni-functionalized oxides exhibit enhanced sorption performance due to strong complexation with oxygen-donor groups and favorable electronic configurations.^25,26^

Motivated by these findings, the present study introduces a novel nanoparticles (GA-NiMMPs) synthesized *via* a green, low-temperature co-precipitation method, combining the biofunctionality of gum Arabic with the magnetic responsiveness of nickel-doped magnetite. To the best of our knowledge, this is one of the first studies to integrate gum Arabic and nickel into a mesoporous magnetite platform and validate its performance in both synthetic and real wastewater matrices. The nanoparticles was extensively characterized to confirm its structure, morphology, and functional surface chemistry. Its adsorption performance was evaluated for Cd(ii) and Cu(ii) ions in single-ion systems, under varying operational parameters, including pH, contact time, adsorbent dosage, and initial ion concentration. Kinetic, isotherm, and thermodynamic models were applied to describe the adsorption mechanisms. The real wastewater matrix used in this study contained multiple heavy metal ions; although dye residues were present, their removal was not quantitatively addressed. This work provides a green synthesis route for a multifunctional, biomass-derived adsorbent, with validated performance in both controlled and real-world treatment scenarios.

## 2. Experiments

### 2.1. Materials

All chemicals used in this study were of analytical grade and were used without further purification. Stock solutions of cadmium (Cd^2+^) and copper (Cu^2+^) ions were prepared using cadmium sulfate octahydrate (CdSO_4_·8H_2_O, ≥99%, Sigma-Aldrich) and copper sulfate pentahydrate (CuSO_4_·5H_2_O, ≥99%, Sigma-Aldrich), respectively, dissolved in double-distilled water. Gum Arabic, ferric chloride hexahydrate (FeCl_3_·6H_2_O), ferrous chloride tetrahydrate (FeCl_2_·4H_2_O), nickel chloride hexahydrate (NiCl_2_·6H_2_O), and ammonium hydroxide (NH_4_OH, 28%) were used for the synthesis of the adsorbent material. These reagents, with a purity of 99.9%, were procured from Sigma-Aldrich, UK. Additionally, high-purity nitric acid (HNO_3_), hydrochloric acid (HCl), and sulfuric acid (H_2_SO_4_) (Merck, Germany) were employed during the desorption process to recover the adsorbed metals and regenerate the adsorbent.

### 2.2. Synthesis of gum Arabic activated magnetite–nickel mesoporous nanoparticles

In this study, gum Arabic-activated magnetite–nickel mesoporous nanoparticles (labeled as GA-NiMMPs) were synthesized *via* a green co-precipitation method adapted from Birniwa *et al.* (2022),^22^ with modifications to incorporate nickel into the magnetite lattice and enhance adsorption capacity for Cd(ii) and Cu(ii) ions. Briefly, an aqueous solution containing 2.0 g of FeCl_2_·4H_2_O, 5.2 g of FeCl_3_·6H_2_O, and 2.0 g of NiCl_2_·6H_2_O was prepared in 100 mL of double-distilled water. The synthesis was carried out as an open batch system at atmospheric pressure. Separately, 2.0 g of gum Arabic was dissolved in 50 mL of double-distilled water under gentle heating and magnetic stirring. This solution was then slowly added to the metal salt mixture under continuous stirring at 80 °C for 30 minutes to ensure homogeneous dispersion. The pH was adjusted to 10 using 28% NH_4_OH solution, initiating co-precipitation and promoting particle growth and stabilization. A black precipitate, characteristic of magnetite formation, was observed. The reaction was maintained at 80 °C for an additional 60 minutes under constant stirring to promote particle growth and stabilization. After cooling to room temperature, the resulting precipitate was separated by centrifugation at 5000 rpm for 10 minutes. The obtained GA-NiMMPs were thoroughly washed with absolute ethanol and double-distilled water to remove unreacted precursors and by-products. The purified product was dried at 60 °C for 12 hours and stored in a desiccator for subsequent characterization and adsorption studies. The complete synthesis sequence, including GA dissolution, metal salt mixing, pH adjustment, magnetic separation, washing, and drying steps—together with the reaction temperature, pH, and stirring conditions—is illustrated in Scheme S1 for clarity.

### 2.3. Sorbent characterization

The physicochemical properties of the synthesized GA-NiMMPs were investigated using a combination of advanced analytical techniques. The surface morphology and microstructure were examined by scanning electron microscopy (SEM; JEOL JSM-6510LV, Japan) operated at an accelerating voltage of 20 kV, following gold sputter-coating of the samples to enhance surface conductivity. Elemental composition and distribution were simultaneously analyzed using energy-dispersive X-ray spectroscopy (EDX) coupled with SEM, which confirmed the presence of Fe, Ni, C, and O and verified successful nickel incorporation and gum Arabic surface functionalization.

Fourier-transform infrared spectroscopy (FTIR) was employed to identify the surface functional groups and bonding interactions. Spectra were recorded in the range of 400–4000 cm^−1^ using a Bruker ALPHA spectrometer in attenuated total reflectance (ATR) mode, revealing characteristic vibrational bands corresponding to hydroxyl (–OH), carboxyl (–COOH), and metal–oxygen bonds (Fe–O and Ni–O). The crystallographic structure of the nanoparticles was investigated *via* X-ray diffraction (XRD) using a PANalytical X'Pert PRO diffractometer equipped with Cu Kα radiation (*λ* = 1.5406 Å), scanned over a 2*θ* range of 10–80°. The resulting diffraction patterns confirmed the presence of magnetite (Fe_3_O_4_), nickel ferrite (NiFe_2_O_4_), and minor NiO phases, indicating successful formation of a mixed-phase spinel structure.

Textural properties, including specific surface area, pore size, and pore volume, were analyzed by nitrogen adsorption–desorption isotherms measured at 77 K using a Micromeritics ASAP 2020 analyzer. The Brunauer–Emmett–Teller (BET) method was used to calculate the surface area, while the Barrett–Joyner–Halenda (BJH) method determined pore size distribution and mesoporosity. Samples were degassed at 150 °C for 12 hours prior to measurement to eliminate moisture and surface contaminants. Additionally, the hydrodynamic particle size and surface charge (zeta potential) of the GA-NiMMPs dispersed in deionized water were determined by dynamic light scattering (DLS) and electrophoretic light scattering using a Malvern Zetasizer Nano ZS. These measurements provided insight into the colloidal stability and electrostatic behavior of the GA-NiMMPs in aqueous environments, which are critical for adsorption performance.

### 2.4. Adsorption experiments

Batch adsorption experiments were conducted to evaluate the efficiency of GA-NiMMPs in adsorbing Cu(ii) and Cd(ii). The experiments were performed using a Thermo-shaker water bath (Scientific Precision SWB 27, Waltham, USA) in polypropylene tubes. Unless otherwise stated, all adsorption experiments were conducted using individual solutions of Cd(ii) or Cu(ii) at an initial concentration of 50 mg L^−1^. A measured mass of adsorbent (*m*, g) was added to a known volume (*V*, L) of the metal-containing solution, and the mixture was agitated on an orbital shaker at 150 rpm for 240 minutes at room temperature (25 ± 1 °C). The effects of solution pH (ranging from 2 to 9) and adsorbent dosage (ranging from 1.0 to 5.0 g L^−1^) were systematically investigated to optimize the adsorption conditions. pH adjustments were made using 0.5 M HCl and 0.5 M NaOH solutions. Desorption studies were carried out to evaluate the reusability of the adsorbents. Metal-loaded samples were treated with 1.0 M solutions of HNO_3_, HCl, or H_2_SO_4_ for 4 hours at an adsorbent dosage of 4.0 g L^−1^. The eluate was analyzed to determine the desorption efficiency.

Adsorption kinetics were studied by varying shaking times (5–600 minutes), while isotherm performance was evaluated at initial metal concentrations of 20–200 mg L^−1^. The Lagergren pseudo-first-order, pseudo-second-order, and intraparticle diffusion (Weber and Morris) models were used to analyze kinetics. The Langmuir, Freundlich, Temkin, and Sips models were applied to describe adsorption isotherms. The non-linear forms of these kinetic and isotherm models are provided in Table S1.^29–34^ The fitting of the models was evaluated based on the Chi-square (*x*^2^) and the coefficient of determination (*R*^2^), as described in Table S1.^33,34^ Thermodynamic parameters, including standard Gibbs free energy change (Δ*G*°), standard enthalpy change (Δ*H*°), and standard entropy change (Δ*S*°), were calculated at temperatures ranging from 25 to 50 ± 1 °C using the equations listed in Table S1.^35,36^ These parameters provided insights into the spontaneity and feasibility of the adsorption process.

In all experiments, the initial (*C*_0_) and final residual (*C*_e_) concentrations of Cu(ii) and Cd(ii) were measured using an Atomic Absorption Spectrometer (GBC 932 AA, UK). Samples were filtered through 0.22 μm filters before analysis. Experiments were conducted in triplicate, and results with a relative error of ≤5% were considered acceptable. The adsorption efficiency (E%), adsorption capacity (*q*_e_, mg g^−1^), and distribution coefficient (*K*_d_) were calculated using the following equations:1
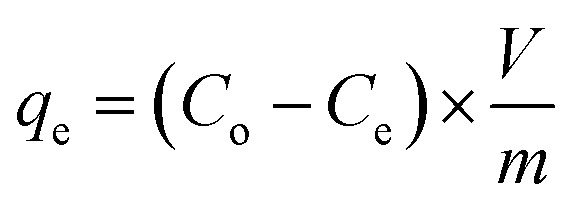
2
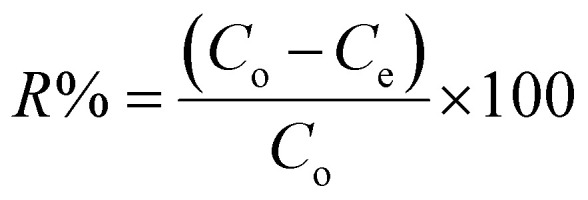
3
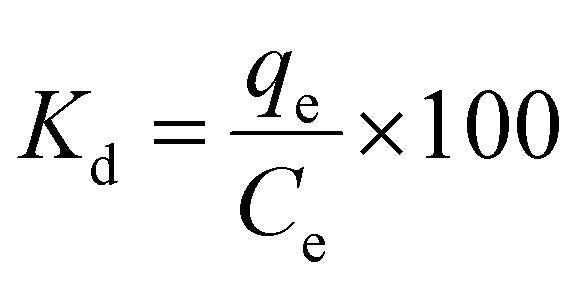


## 3. Results and discussion

Developing efficient, green-engineered materials for heavy metal removal is a key step toward sustainable water treatment solutions. In this study, GA-NiMMPs was synthesized and evaluated for its performance in removing Cd(ii) and Cu(ii) ions from aqueous environments. The following sections present a comprehensive investigation of the material's structural characteristics, surface properties, adsorption behavior, and regeneration potential. Each result is discussed in the context of its relevance to real-world application, emphasizing how the incorporation of nickel enhances both functional performance and selectivity in complex water matrices.

### 3.1. Characterization of GA-NiMMPs

#### 3.1.1. Structural composition & chemical identification

**Fig. 1:**
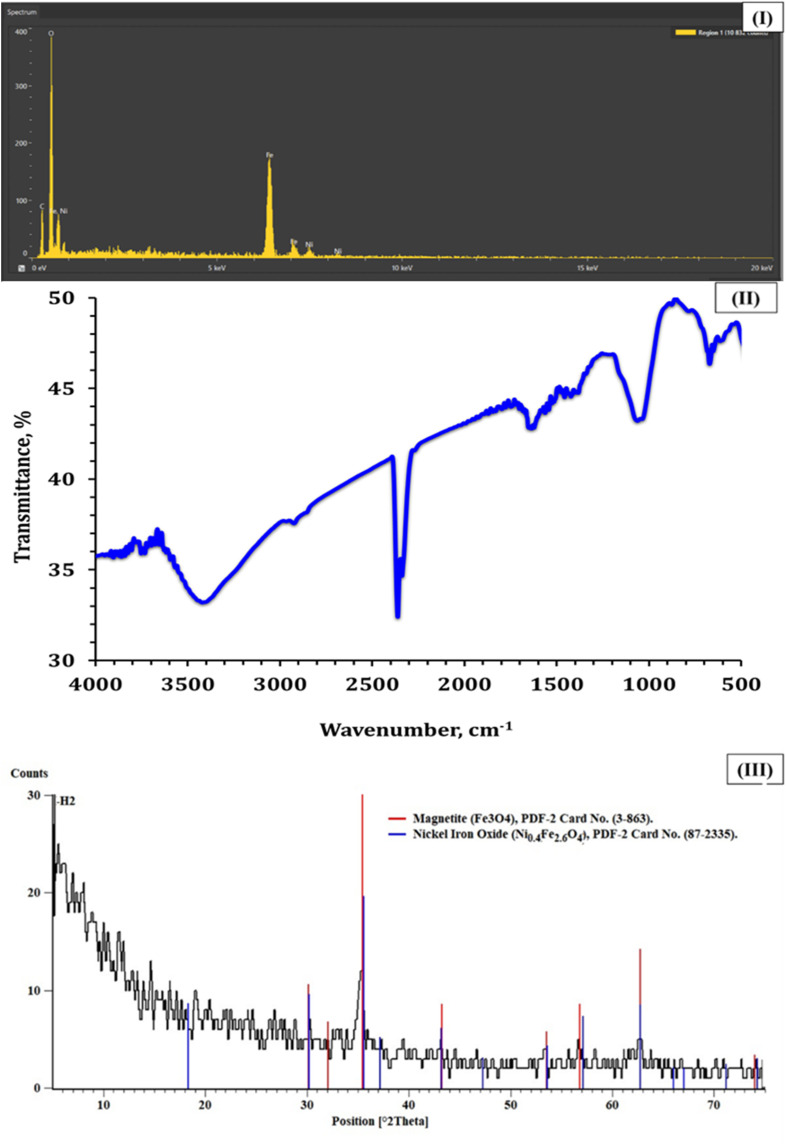
EDX spectrum (I), IR spectrum (II), and XRD pattern (III) of the GA-NiMMPs sorbent.

Energy-dispersive X-ray spectroscopy (EDX) revealed the elemental constitution of the GA-NiMMPs, identifying Fe, Ni, O, and C as the principal elements (Fig. 1-I). The prominent oxygen peak confirmed extensive surface oxidation, indicative of mixed metal oxide formation, including magnetite (Fe_3_O_4_) and nickel-substituted ferrite phases (Ni_0_._4_Fe_2_._6_O_4_). This oxidative environment supports the generation of reactive oxygen species and surface functionalities critical for metal ion sorption.^25,36^ Nickel incorporation into the Fe_3_O_4_ lattice enhances redox capability and introduces lattice distortions that potentially create additional adsorption-active sites.^25,26^ Importantly, the EDX spectrum exhibited a clear carbon signal (C ∼8.2 wt%), which cannot originate from inorganic precursors and thus verifies the presence of gum Arabic (GA) on the composite surface. This is consistent with the separate EDX analysis of pure GA reported recently,^37^ where GA displayed a dominant carbon peak alongside minor oxygen contributions, reflecting its polysaccharide nature. The comparison confirms that the carbon detected in GA-NiMMPs arises from GA coating and not from contamination or background noise. Together with the FTIR results (bands at ∼1700 cm^−1^, 1400 cm^−1^, and 1250 cm^−1^), this provides strong evidence of effective surface functionalization by GA. In addition to confirming GA functionalization, the simultaneous detection of Fe, Ni, and O corroborates the formation of mixed ferrite phases, which are intrinsically magnetic. These findings, combined with XRD confirmation of Fe_3_O_4_ and NiFe_2_O_4_ crystalline phases, strongly support the magnetic nature of the synthesized GA-NiMMPs.

The co-precipitation mechanism involves the hydrolysis of Fe^3+^, Fe^2+^, and Ni^2+^ ions in the presence of ammonium hydroxide, leading to the *in situ* formation of mixed metal hydroxides. Upon heating and aging, these hydroxides transform into a bimetallic spinel structure composed of magnetite (Fe_3_O_4_) and nickel ferrite (NiFe_2_O_4_). The alkaline pH (∼10) facilitates the formation of Ni(OH)_2_, which subsequently integrates into the Fe–O lattice during the growth process. This mechanism is consistent with the XRD results, confirming crystalline phases of both Fe_3_O_4_ and NiFe_2_O_4_. GA therefore plays a dual role—acting both as a reducing and stabilizing agent during synthesis and as a functional surface modifier post-synthesis. It introduces hydroxyl (–OH) and carboxyl (–COOH) functional groups, which are key for complexation and electrostatic interaction with divalent metal ions.^25,36^ This surface functionalization also facilitates water dispersibility and colloidal stability, crucial attributes for sorbent performance in aqueous environments. The elemental composition measured by EDX (Fe ∼64.23%, Ni ∼6.41%, O ∼21.61%, and C ∼8.2 wt%) thus validates the successful incorporation of GA and its functional role in modifying the composite surface.

Fourier transform infrared (FTIR) spectra (Fig. 1-II) provide clear evidence of the chemical functionalities present on the GA-NiMMPs surface. The distinct absorption band observed between 550–600 cm^−1^ corresponds to Fe–O stretching vibrations, confirming the formation of magnetite and nickel ferrite phases.^22–24^ The broad band centered at ∼3400 cm^−1^ is attributed to O–H stretching vibrations of hydroxyl groups and adsorbed water molecules,^22–24^ which are crucial for surface complexation and hydrogen bonding during adsorption. Well-defined peaks at ∼1700 cm^−1^ (C=O stretching) and ∼1400 cm^−1^ (COO^−^ symmetric stretching) confirm the incorporation of carboxyl functionalities derived from gum Arabic, serving as strong binding sites for Cd(ii) and Cu(ii). A weaker shoulder at ∼1250 cm^−1^ (C–O stretching), together with the feature around 1375 cm^−1^ (C–H bending), further demonstrates the retention of polysaccharide groups from GA on the composite surface.^22–24^ These functional moieties enable multiple adsorption pathways including electrostatic attraction, ion exchange, and hydrogen bonding, enhancing the versatility and binding specificity of the GA-NiMMPs.

X-ray diffraction (XRD) analysis (Fig. 1-III) revealed sharp diffraction peaks at 2*θ* = 30.1°, 35.5°, 43.2°, 53.5°, and 62.7°, corresponding to the (220), (311), (400), (422), and (511) planes of Fe_3_O_4_ (JCPDS 19-0629), confirming magnetite as the predominant phase.^22–24^ Superimposed reflections at 37.2° and 57.5° correspond to Ni-ferrite (Ni_0_._4_Fe_2_._6_O_4_) (JCPDS 10-0325), validating nickel integration within the spinel structure.^25,26^ Additionally, minor peaks corresponding to NiO (JCPDS 47-1049) suggest partial segregation of Ni as a discrete oxide phase, likely contributing to enhanced electron transport and sorption dynamics.^25,26^ Broadening of the XRD peaks, particularly at 35.5° and 43.2°, indicates nanoscale crystallite domains and possible lattice strain resulting from metal ion substitution and oxygen vacancy formation. These crystalline and sub-crystalline features enhance the number of active surface sites and increase the reactivity of the sorbent.^25,26^ Partial oxidation of Fe_3_O_4_ to maghemite may have occurred during synthesis or post-processing, further influencing the electronic configuration of the surface. Collectively, the hybrid crystalline structure comprising Fe_3_O_4_, Ni-ferrite, and NiO contributes to structural robustness, magnetic responsiveness, and enhanced metal ion affinity.

#### 3.1.2. Morphological & surface characteristics

**Fig. 2:**
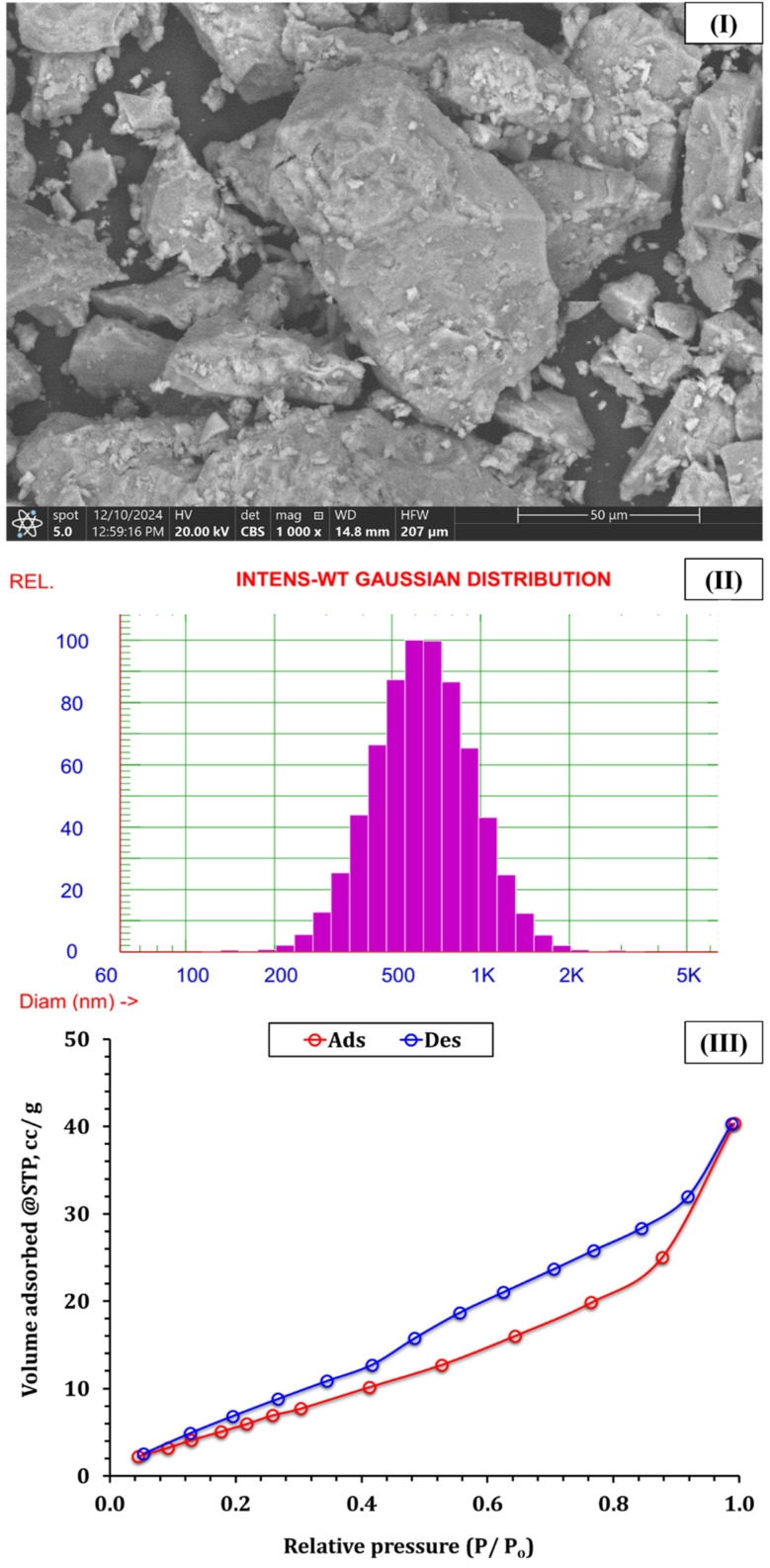
(I) SEM image of GA–NiMMPs, (II) DLS particle size distribution, and (III) nitrogen adsorption–desorption isotherm.

Scanning electron microscopy (SEM) images (Fig. 2-I) revealed non-uniform, irregularly shaped particles with porous and rugged surfaces. These morphological features are desirable in adsorbent materials, as they increase the available surface area and enhance the accessibility of active binding sites.^22–24^ The observed surface roughness and pore networks suggest a mesoporous framework, which is essential for facilitating rapid intraparticle diffusion of metal ions. The presence of a GA coating was evident in the micrographs, contributing to uniform nanoparticle distribution and suppressing aggregation.^25,26^ This biomolecular layer not only stabilizes the particle dispersion but also enhances functional surface group exposure, thereby improving adsorption efficiency. The combined morphological characteristics confirm that the composite has a hybrid architecture, with bio-organic and inorganic constituents working synergistically to enhance performance.

Dynamic light scattering (DLS) analysis (Fig. 2-II and Table 1) revealed an average hydrodynamic diameter of 688.3 nm, with 75% of the particles measuring below 828.5 nm. The presence of a broad size distribution suggests that while most particles are in the sub-micron range, some degree of agglomeration may have occurred due to the nature of bio-assisted synthesis. Nevertheless, the average particle size supports a favorable surface area-to-volume ratio, which is critical for adsorption kinetics.^22–24^ The particle size results align with SEM observations (Fig. 2-II) and confirm that the GA-NiMNPs exhibit a well-dispersed, moderately polydisperse morphology. The relatively small size ensures efficient diffusion and interaction with metal ions in aqueous environments. Moreover, the size distribution may be attributed to lattice distortion induced by Ni doping and surface functionalization, highlighting the influence of synthesis parameters on final particle characteristics.

**Table 1:** DLS and zeta potential values of the prepared ligand

DLS analysis, nm	Zeta potential, mV
688.3	−21.4

Zeta potential measurements indicated a surface charge of −21.32 mV (Table 1), reflecting moderate colloidal stability and a net negative surface potential. This negative charge results from the deprotonation of carboxyl and hydroxyl groups contributed by GA, as confirmed by FTIR analysis (Fig. 1-II). It facilitates electrostatic attraction of positively charged heavy metal ions, enhancing the adsorption capacity of the GA-NiMMPs.^22–24^ This moderately negative zeta potential also helps prevent aggregation by promoting electrostatic repulsion among particles in suspension, preserving a high degree of dispersion in aqueous environments.^22–24^ Together with the porous structure and small particle size, this surface potential supports efficient ion accessibility and high sorption efficiency, further validating the functional role of GA in enhancing material stability and performance.

Nitrogen adsorption–desorption isotherms (Fig. 2-III) exhibited a Type IV pattern, according to the IUPAC classification with a pronounced H3 hysteresis loop at relative pressures (*P*/*P*_0_) > 0.4, characteristic of mesoporous materials.^27^ This profile suggests the existence of slit-shaped pores and indicates well-defined mesostructures, often formed by the stacking of plate-like particles or irregular pore assemblies. The BET surface area of 29.32 m^2^ g^−1^ demonstrates a moderately high external surface area, sufficient to support rapid metal ion uptake. The *C* constant value of 7.48 reflects favorable interactions between nitrogen molecules and the composite surface, implying a high density of adsorption sites. This value correlates with the presence of oxygen-containing functional groups and the surface roughness observed in SEM images. The porosity, in conjunction with surface chemistry, creates an ideal microenvironment for adsorption processes, especially in aqueous-phase applications. BJH pore analysis (Fig. S2) confirmed the mesoporous nature of the GA-NiMNPs, showing an average pore radius of 1.91 nm and a total pore volume of 0.0577 cm^3^ g^−1^. The cumulative pore volume, as determined by DFT, was 0.0624 cm^3^ g^−1^, with a pore width distribution primarily in the 2–4 nm range. These properties are summarized in Table 2. These results affirm the hierarchical mesoporous framework, which enables effective diffusion and retention of metal ions. Such a pore structure ensures that the material maintains a balance between surface area and pore accessibility, essential for adsorption under dynamic flow conditions. The mesopores, supported by GA-induced particle spacing and nickel-enhanced lattice structuring, contribute to the GA-NiMMPs overall adsorption kinetics and equilibrium behavior. This configuration promotes not only rapid metal capture but also ease of regeneration, enhancing the reusability of the biomass-derived sorbent for environmental applications.

**Table 2:** Summary of textural properties of GA-NiMMPs sorbent

*S*_BET_ (m^2^ g^−1^)	*V*_P_ (cm^3^ g^−1^)	*D*_H_ (nm)
29.32	0.0624	1.91

### 3.2. Batch investigation

**Fig. 3:**
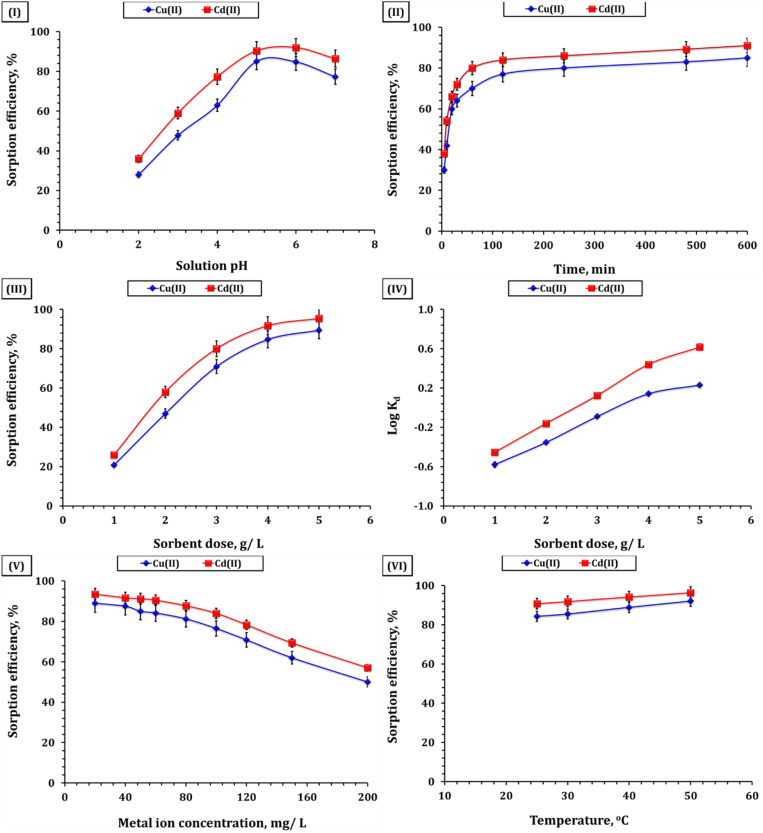
Sorption efficiency of copper(ii), and cadmium(ii) depending on (I) solution pH (GA-NiMMPs dosage: 4.0 g L^−1^; initial concentration: 50 mg L^−1^; room temperature; and time: 240 min), (II) shaking time (room temperature; GA-NiMMPs dosage of 4.0 g L^−1^; initial concentration of 50 mg L^−1^; pH 5.9), (III) ligand dosage (pH 5.9; time of 240 min; initial concentration: 50 mg L^−1^; and room temperature), (IV) distribution coefficient (log *K*_d_) on GA-NiMMPs dose (pH 5.9; time of 240 min; initial concentration: 50 mg L^−1^; and room temperature), (V) initial concentration (time: 240 min; room temperature; pH 5.9; and GA-NiMMPs dosage: 4.0 g L^−1^), and (VI) temperature (pH 5.9; time of 240 min; initial concentration: 50 mg L^−1^; GA-NiMMPs dosage: 4.0 g L^−1^).

The adsorption performance of GA-NiMMPs for Cd(ii) and Cu(ii) ions was systematically evaluated under varying conditions, including pH, contact time, adsorbent dosage, initial metal ion concentration, and temperature. The results demonstrate high adsorption efficiency for Cd(ii) and Cu(ii) across a range of conditions, with maximum removal exceeding 96% under optimal pH, dosage, and contact time (Fig. 3).

Solution pH plays a pivotal role in adsorption behavior by modulating both the ionization state of surface functional groups on the sorbent and the speciation of metal ions in solution.^11–14^ As shown in Fig. 3-I, the adsorption efficiency of GA-NiMMPs for Cd(ii) and Cu(ii) increased progressively with rising pH and reached a maximum at pH 5–6, where removal efficiencies were 90.4% for Cd(ii) and 85.1% for Cu(ii). This trend reflects the combined influence of surface charge characteristics and metal ion speciation. At strongly acidic pH values (2–3), extensive protonation of carboxyl and hydroxyl groups on gum Arabic suppresses their ability to coordinate with Cd(ii) and Cu(ii), while the zeta potential measurement (−21.32 mV) further indicates reduced electrostatic attraction under such proton-rich conditions. At near-neutral pH (5–6), deprotonation of –COOH and –OH groups increases the density of negatively charged sites, which enhances electrostatic attraction toward the divalent cations (Cd^2+^, Cu^2+^). In addition, surface Ni–O and Fe–O sites originating from the spinel lattice of Ni-ferrite and magnetite play a direct role by acting as electron-donor centers, enabling inner-sphere complexation with the metal ions. The synergistic effect of GA-derived functional groups and lattice metal–oxygen sites explains the high adsorption performance observed in this range. This interpretation is fully consistent with FTIR spectra (Fig. 1-II), which highlight the presence of carboxylate and hydroxyl functionalities, as well as with XRD evidence confirming Ni incorporation into the Fe_3_O_4_ structure. At alkaline pH values above 7, the efficiency declined, which can be attributed to the onset of metal hydroxide precipitation (Cd(OH)_2_ and Cu(OH)_2_). Under these conditions, the removal is no longer governed solely by adsorption but also by precipitation processes, making the measured uptake less representative of surface binding.^15–18^ Therefore, the optimal adsorption window for GA-NiMMPs lies between pH 5 and 6, where the combined contribution of GA functional groups and Ni/Fe oxide lattice sites maximizes electrostatic attraction and coordination with Cd(ii) and Cu(ii) ions.^20–22^

Adsorption kinetics showed a rapid uptake during the initial 120 minutes, where removal efficiencies reached 84.0% for Cd(ii) and 77.0% for Cu(ii), and gradually approached equilibrium after 600 minutes, stabilizing at 91.0% and 84.9%, respectively (Fig. 3-II). The fast initial phase reflects the abundance of unoccupied active sites and the accessibility provided by the mesoporous structure.^10–12^ As equilibrium is approached, the adsorption rate slows due to site saturation,^14–16^ which is supported by the material's high surface area and mesoporous structure (BET surface area = 29.32 m^2^ g^−1^; Table 2), providing accessible pathways for initial rapid uptake followed by diffusion-limited behavior.

The adsorption efficiency improved significantly with increasing GA-NiMNPs dosage, reaching a plateau at 5.0 g L^−1^, with Cd(ii) and Cu(ii) removal efficiencies of 96.4% and 92.1%, respectively (Fig. 3-III). The increase in removal corresponds to the greater availability of active binding sites at higher dosages.^10–12^ Beyond 4.0 g L^−1^, only marginal gains were observed, suggesting near-saturation of the system.^14–16^ This saturation was further validated through changes in the partition coefficient (log *K*_d_). At low dosage (1.0 g L^−1^), the log *K*_d_ values were negative, reflecting poor adsorption due to limited sites.^14–16^ However, values turned positive at 4.0 g L^−1^ (0.44 for Cd(ii) and 0.14 for Cu(ii)), confirming enhanced metal uptake (Fig. 3-IV).^14–16^ The difference in Cd(ii) and Cu(ii) partitioning reflects their respective physicochemical behaviors and affinity for the sorbent.

Initial concentrations ranging from 20 to 200 mg L^−1^ revealed that GA-NiMMPs perform exceptionally well at low to moderate concentrations, achieving removal efficiencies of 93.5% for Cd(ii) and 89.0% for Cu(ii) at 20 mg L^−1^ (Fig. 3-V). At higher concentrations, efficiency declined due to site saturation, with values decreasing to 57.0% (Cd) and 50.0% (Cu) at 200 mg L^−1^. This trend reflects a capacity-limited system in which the abundance of available sites at 20–50 mg L^−1^ ensures high binding, while saturation dominates at higher loadings.^10–12^ Correspondingly, the adsorption capacity (*q*_e_) increased steadily with initial concentration: from 4.7 mg per g (Cd) and 4.5 mg per g (Cu) at 20 mg L^−1^, to 11.4 mg per g (Cd) and 10.6 mg per g (Cu) at 50 mg L^−1^. Beyond this point, *q*_e_ continued to rise with increasing concentration, reaching 28.5 mg per g (Cd) and 25.0 mg per g (Cu) at 200 mg L^−1^, although with lower removal efficiency. Therefore, 50 mg L^−1^ was selected as the optimum initial concentration, since it simultaneously provided high removal efficiencies (>90%) and the maximum adsorption capacity observed under the studied conditions (11.4 mg g^−1^ for Cd and 10.6 mg g^−1^ for Cu). The structural features of GA-NiMMPs—including mesoporosity and abundant surface functional groups—aid in sustaining performance under moderate loading, while capacity-driven binding dominates at higher concentrations.

Temperature-dependent adsorption tests showed a marked increase in efficiency with rising temperature (25–50 °C), confirming the endothermic nature of the process.^10–12^ For Cu(ii), efficiency rose from 84.3% to 92.1%, and for Cd(ii), from 90.8% to 96.4% (Fig. 3-VI). This enhancement is attributed to increased kinetic energy and reduced hydration shell size, facilitating stronger sorbate–sorbent interactions.^10–12^ FTIR and XRD analysis (Fig. 1-II and III respectively) support that higher temperatures enhance functional group reactivity and promote stronger complexation.^14–16^ These results highlight the suitability of GA-NiMNPs for thermally variable environments and industrial effluents.

Collectively, these findings confirm that GA-NiMNPs are highly efficient, biomass-derived adsorbents for Cd(ii) and Cu(ii), with performance directly tied to their structural, surface, and textural attributes. The synergy between porosity, surface charge, and bio-functionalization facilitates multi-modal interactions—electrostatic, coordinative, and diffusional—offering a robust platform for sustainable water purification. Similar adsorption performance for copper(ii) and cadmium(ii) ions from aqueous solutions has been observed using various adsorbents, including CuO-modified ceramic membranes,^4^ chitosan–vermiculite composites,^11^ Ag-MOF/chitosan composite sponges,^12^ exhausted copper slag-supported sulfidized nanoscale zerovalent iron,^14^ dithiocarbamate-functionalized activated carbon,^15^*Cystoseira sedoide* algae,^16^ and ethylenediamine-functionalized chelating resin 28. These findings provide valuable insights into optimizing process conditions and highlight the potential of this adsorbent for practical applications in wastewater treatment and environmental remediation, particularly in complex aqueous matrices.

### 3.3. Sorption kinetic investigation

**Fig. 4:**
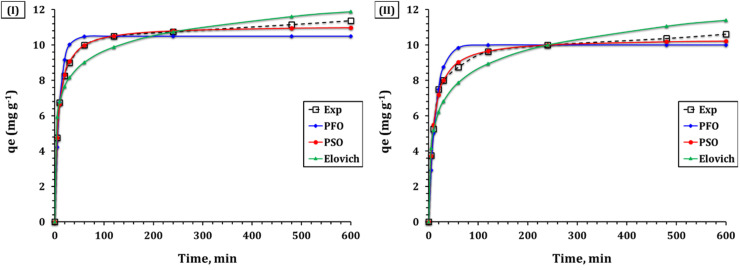
Kinetic model fitting for the adsorption of (I) Cd(ii) and (II) Cu(ii) onto GA–NiMMPs at solution pH 6.01, adsorbent dosage of 4.0 g L^−1^, initial concentration of 50 mg L^−1^, and temperature of 25 ± 1 °C.

The kinetic investigation of Cd(ii) and Cu(ii) adsorption on GA-NiMMPs provides critical insights into the adsorption mechanism, rate-limiting steps, and the influence of the material's physicochemical properties on the adsorption process.^29,30^ To evaluate the experimental data, we applied several conventional kinetic models, including Morris–Weber, Elovich, pseudo-second-order (PSO), and pseudo-first-order (PFO). The kinetic curves for Cd(ii) and Cu(ii) adsorption are illustrated in Fig. 4, and the parameters of the applied kinetic models are summarized in Table 3.

**Table 3:** The values of the parameters used in the applied kinetics model

	Cd(ii)	Cu(ii)
**Pseudo first-order model**		
*q*_1_ (mg g^−1^)	10.5	10.0
*k*_1_ (min^−1^)	0.103	0.069
*R* ^2^	0.91	0.95
*X* ^2^	0.40	0.49

**Pseudo second-order model**		
*q*_2_ (mg g^−1^)	11.1	10.4
*k*_2_ (min^−1^)	0.013	0.011
*h* (mol g^−1^ h^−1^)	1.7	1.2
*t*_1/2_ (h)	6.7	8.9
*R* ^2^	0.99	0.99
*X* ^2^	0.02	0.05

**Elovich model**		
*a* (mg g^−1^ min^−1^)	0.80	0.65
*β* (g mg^−1^)	29.08	4.32
*R* ^2^	0.90	0.91
*X* ^2^	0.6	0.8

The pseudo-first-order model yielded relatively lower correlation coefficients (*R*^2^ = 0.91 for Cd(ii) and 0.95 for Cu(ii)) and higher error values (*χ*^2^ = 0.40 for Cd(ii) and 0.49 for Cu(ii)) (Table 3), indicating limited applicability for these systems. In contrast, the pseudo-second-order model exhibited an excellent fit, with *R*^2^ values of 0.99 for both Cd(ii) and Cu(ii) and significantly lower error values (*χ*^2^ = 0.02 for Cd(ii) and 0.05 for Cu(ii)). The superior fit of this model suggests that chemisorption governs the adsorption process, involving strong chemical interactions between the metal ions and the functional groups on the GA-NiMMPs surface.^11–15^ These kinetic trends align well with findings from FTIR analysis (Fig. 1-II), which confirms the presence of –OH, –COO^−^, and –C=O moieties on the GA-NiMMPs surface. This validation reinforces the chemisorptive nature of the adsorption system, highlighting the role of specific functional groups in the binding process. Furthermore, the pseudo-second-order model also revealed higher adsorption capacities (*q*_2_ = 11.1 mg g^−1^ for Cd(ii) and 10.4 mg g^−1^ for Cu(ii)), consistent with the experimental data. The rate constants (*k*_2_ = 0.013 min^−1^ for Cd(ii) and 0.011 min^−1^ for Cu(ii)), the initial sorption rates (*h* = 1.7 mol g^−1^ h^−1^ for Cd(ii) and 1.2 mol g^−1^ h^−1^ for Cu(ii)), and half-life values (*t*_1_/_2_ = 6.7 h for Cd(ii) and 8.9 h for Cu(ii)) suggest a relatively fast adsorption process, with Cd(ii) being adsorbed slightly faster than Cu(ii). This difference is likely due to the smaller ionic radius and lower hydration energy of Cd(ii), which facilitates faster diffusion and stronger binding.^10,12^

The Elovich model, which describes chemisorption on highly heterogeneous surfaces, also provided a good fit to the experimental data, with *R*^2^ values of 0.90 for Cd(ii) and 0.91 for Cu(ii). The high initial adsorption rate (*α* = 0.80 mg g^−1^ min^−1^ for Cd(ii) and 0.65 mg g^−1^ min^−1^ for Cu(ii)) and the desorption constant (*β* = 29.08 g mg^−1^ for Cd(ii) and 4.32 g mg^−1^ for Cu(ii)) suggest that as evidenced by the variation in pore size and surface morphology seen in SEM and BET data. Such physical heterogeneity is consistent with Elovich assumptions of chemisorption across energetically diverse sites.^10,12,38^ This matches well with the high surface roughness and pore diversity observed *via* SEM and BET analysis (Fig. 2-I, and Table 2).

**Fig. 5:**
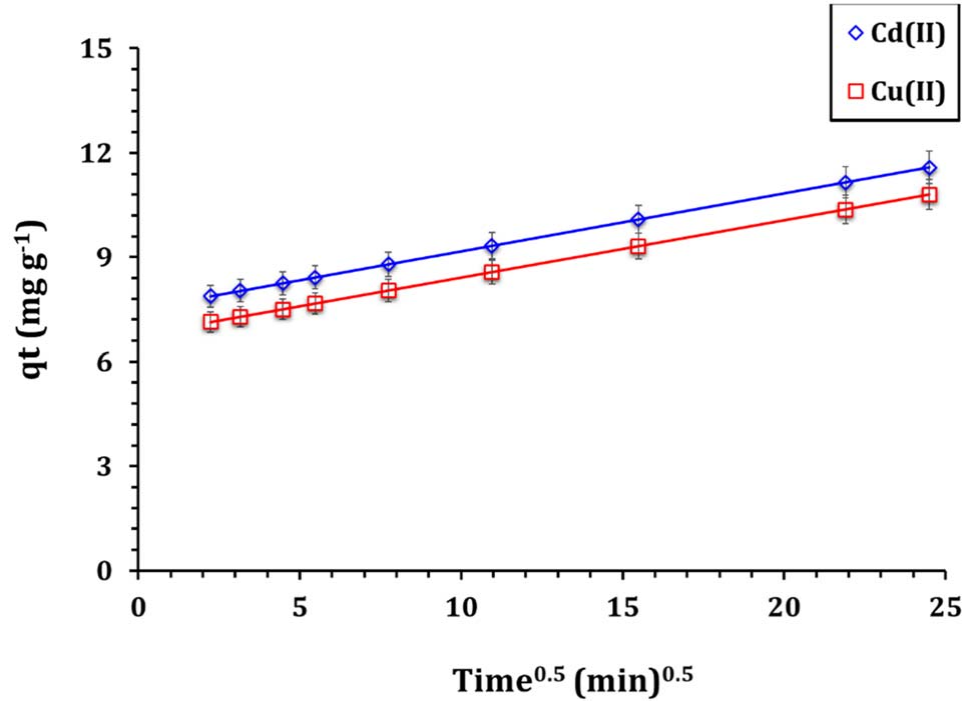
W–M model illustrate for uptake process (solution pH 6.01; ligand dose of 4.0 g L^−1^; original concentration of 50 mg L^−1^; temperature of 25 ± 1 °C).

The Weber–Morris model indicates that adsorption follows a multi-step pathway, beginning with external surface adsorption, followed by intraparticle diffusion, and finally reaching equilibrium.^11–15^ The initial stage, characterized by a steep slope, signifies the rapid adsorption of metal ions onto the external surface of GA-NiMMPs. In the second stage, metal ions gradually diffuse into the interior pores of the adsorbent. The final stage represents equilibrium, at which point the adsorption rate slows due to site saturation.^11–15^ The Weber–Morris model parameters (Table 4) reveal that the intraparticle diffusion rate constants (*k*_i_ = 0.16 mg g^−1^ min^−1/2^ for Cd(ii) and Cu(ii)) and the intercept values (*C* = 7.5 for Cd(ii) and 6.8 for Cu(ii)) suggest that intraparticle diffusion is not the only rate-limiting step. These findings, when combined with the high correlation of the pseudo-second-order model and the nonlinear *qt vs. t*^1^/^2^ plot (Fig. 5), confirm that adsorption occurs *via* a dual mechanism involving both surface adsorption and intraparticle diffusion. The high *R*^2^ values (0.98) affirm the model's applicability, and the multi-linearity of the *qt vs. t*^1^/^2^ plots (Fig. 5) supports this interpretation. These results underscore that the adsorption mechanism is primarily chemisorptive, supplemented by diffusion and governed by the sorbent's heterogeneous mesoporous network.

**Table 4:** The values of Morris–Weber model parameters

		Cd(ii)	Cu(ii)
Weber and Morris model	*k*_i_ (mg g^−1^ min^−1/2^)	0.16	0.16
	*C*	7.5	6.8
	*R* ^2^	0.98	0.98

Overall, the kinetic evaluation underscores the effectiveness of GA-NiMNPs in enabling rapid and efficient metal ion removal. The fast initial adsorption, governed by abundant functional sites, is complemented by a robust porous framework that facilitates prolonged interaction and diffusion. These dual characteristics—chemical specificity through GA-derived functional groups and mesoporosity through nanoarchitectural control—demonstrate a highly synergistic adsorption process. This mechanistic clarity enhances the material's application potential, particularly for dynamic water treatment systems where contact time and efficiency are critical performance parameters. Comparable kinetic patterns have been documented for copper(ii) and cadmium(ii) ion sequestration across diverse adsorbent systems, including CuO-modified ceramic membranes,^4^ chitosan–vermiculite composites,^11^ Ag-MOF/chitosan composite sponges,^12^ exhausted copper slag-supported sulfidized nanoscale zerovalent iron,^14^ dithiocarbamate-functionalized activated carbon,^15^*Cystoseira sedoide* algae,^16^ and ethylenediamine-functionalized chelating resin 28.

### 3.4. Sorption isotherms

**Fig. 6:**
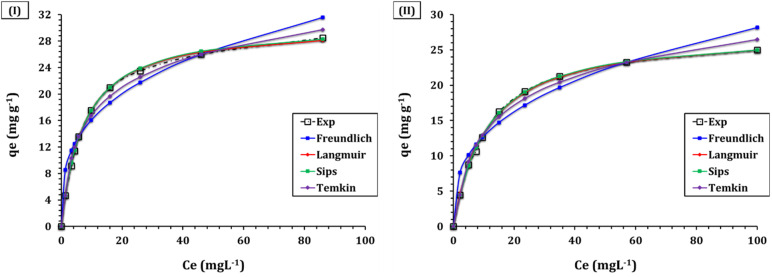
Isotherm profile for the sorption of (I) Cd(ii) and (II) Cu(ii) ions from aqueous onto GA–NiMMPs (time is 240 min; ligand dose of 4.0 g L^−1^; pH 5.9; temperature of 25 ± 1 °C).

The isotherm investigation of Cd(ii) and Cu(ii) adsorption on GA-NiMMPs provides critical insights into the adsorption capacity, affinity, and mechanisms governing the interaction between the metal ions and the adsorbent surface.^31–33^ The isotherm profiles for Cd(ii) and Cu(ii) adsorption are illustrated in Fig. 6, and the parameters of the applied isotherm models are summarized in Table 5.

**Table 5:** Parameters of the applied isotherm models

	Cd(ii)	Cu(ii)
**Langmuir model**		
*q*_m_ (mg g^−1^)	30.4	27.7
*K*_L_ (L mg^−1^)	0.139	0.092
*R* ^2^	0.99	0.99
*X* ^2^	0.04	0.06

**Freundlich model**		
1/*n*_F_	0.3	0.3
*k*_F_ (mg g^−1^) (mg L^−1^)	7.9	5.8
*R* ^2^	0.93	0.92
*X* ^2^	3.2	2.5

**Temkin model**		
*b*_T_ (J mol^−1^)	414.3	428.9
*A*_T_ (L g^−1^)	1.7	1.0
*R* ^2^	0.99	0.99
*X* ^2^	0.4	0.3

**Sips model**		
*q*_S_ (mg g^−1^)	30.8	27.5
*k*_S_ (L mg^−1^)	0.135	0.094
*m*_S_	0.99	1.04
*R* ^2^	0.99	0.99
*X* ^2^	0.04	0.04

The Langmuir model, which assumes monolayer adsorption on a homogeneous surface with a finite number of identical sites, provided an excellent fit to the experimental data, with *R*^2^ values of 0.99 for both Cd(ii) and Cu(ii). The maximum monolayer adsorption capacities (*q*_m_) were 30.4 mg g^−1^ for Cd(ii) and 27.7 mg g^−1^ for Cu(ii), consistent with the high density of accessible binding sites inferred from BET and SEM analysis. The Langmuir constant (*k*_L_), which reflects the adsorption affinity, was higher for Cd(ii) (0.139 L mg^−1^) compared to Cu(ii) (0.092 L mg^−1^), suggesting stronger interactions between Cd(ii) ions and the adsorbent surface.^10,15,16,36^ These results validate the homogenous nature of binding sites provided by GA-functionalized magnetic nanoparticles and support strong electrostatic and complexation interactions. Comparable isotherm behavior, characterized by homogeneous and monolayer adsorption processes, was observed for the removal of copper(ii), and cadmium(ii) from aqueous solutions using various adsorbents. These include CuO-modified ceramic membranes,^4^ chitosan–vermiculite composites,^11^ Ag-MOF/chitosan composite sponges,^12^ exhausted copper slag-supported sulfidized nanoscale zerovalent iron,^14^ dithiocarbamate-functionalized activated carbon,^15^*Cystoseira sedoide* algae,^16^ and ethylenediamine-functionalized chelating resin.^28^ The nature of the sorption system can be assessed using the dimensionless equilibrium parameter (*R*_L_), defined by the equation *R*_L_ = 1/(1 + *K*_L_*C*_0_). This variable classifies sorption behavior into distinct categories: linear (*R*_L_ = 1), irreversible (*R*_L_ = 0), favorable (0 < *R*_L_ < 1), or unfavorable (*R*_L_ > 1).^33,34^ As demonstrated in Fig. S1, the obtained *R*_l_ values confirm that the sorption process is favorable. Specifically, Cd(ii) exhibited *R*_L_ values ranging from 0.03 to 0.26, while Cu(ii) showed values between 0.05 and 0.35 across all tested initial concentrations.

The Freundlich model, which describes adsorption on heterogeneous surfaces with non-uniform energy distribution, also provided a good fit to the experimental data, with *R*^2^ values of 0.93 for Cd(ii) and 0.92 for Cu(ii). The Freundlich constant (*k*_F_), which indicates adsorption capacity, was higher for Cd(ii) (7.9 mg g^−1^) compared to Cu(ii) (5.8 mg g^−1^), further supporting the stronger affinity of GA-NiMMPs for Cd(ii) ions. The heterogeneity factor (1/*n*_F_) was 0.3 for both metal ions, indicating favorable adsorption conditions and a high degree of surface heterogeneity.^31–33^ This matches the Elovich and Weber–Morris model results and is supported by the presence of diverse surface functionalities.^10,15,16,36^ The Temkin model, which accounts for the effects of adsorbate–adsorbent interactions on adsorption heat, provided a strong fit to the experimental data, with *R*^2^ values of 0.99 for both Cd(ii) and Cu(ii). The Temkin heat of adsorption (*b*_T_ = 414.3 J mol^−1^ for Cd(ii), 428.9 J mol^−1^ for Cu(ii)) and equilibrium binding constants (*A*_T_ = 1.7 L g^−1^ for Cd(ii), 1.0 L g^−1^ for Cu(ii)) suggest moderate physisorption alongside chemisorption, particularly at higher concentrations where multilayer interactions are more likely.^12,15,16,29^

Lastly, Sips model, which combines the features of the Langmuir and Freundlich models, provided an excellent fit to the experimental data, with *R*^2^ values of 0.99 for both Cd(ii) and Cu(ii). The maximum adsorption capacities (*q*_S_) were 30.8 mg g^−1^ for Cd(ii) and 27.5 mg g^−1^ for Cu(ii), aligned with Langmuir values. The Sips constant (*k*_S_) was higher for Cd(ii) (0.135 L mg^−1^) compared to Cu(ii) (0.094 L mg^−1^), further supporting the stronger affinity of GA-NiMMPs for Cd(ii) ions. The heterogeneity parameter (*m*_S_) was close to 1.0 for both metal ions, affirmed a predominantly monolayer mechanism, consistent with the Langmuir model.^31,32^ These findings collectively demonstrate that adsorption occurs *via* both homogeneous and heterogeneous interactions, consistent with the material's structural and surface features.

Overall, the isotherm modeling results provide a comprehensive understanding of the adsorption mechanism, validating the structural-functional synergy of GA-NiMMPs. The strong agreement with the Langmuir model highlights the dominance of monolayer coverage on well-defined active sites, while the satisfactory fit with Freundlich and Sips models acknowledges the surface heterogeneity introduced by gum Arabic functionalization. Additionally, Temkin analysis supports moderate interaction energies, suggesting the involvement of both physical and chemical sorption processes. This multipronged adsorption behavior aligns with the hybrid composition of the sorbent, where the natural polymer framework, transition metal oxide phases, and mesoporous architecture collectively influence performance. These findings are further reinforced by the Weber–Morris kinetic model, which reveals a complex interplay of binding modes. The material's effectiveness can be attributed to its mesoporous structure, high surface area, and functionalized chemistry—particularly the presence of key functional groups (–COOH, –OH, and –C=O) that facilitate chemisorption, a negatively charged surface promoting electrostatic interactions, and a porous structure providing extensive binding sites. Together, these properties establish GA-NiMMPs as an efficient and robust adsorbent for heavy metal removal, offering valuable insights for the development of advanced, sustainable water treatment materials.

The higher adsorption tendency of GA-NiMMPs for Cd(ii) over Cu(ii) arises from a combination of physicochemical properties and coordination chemistry principles. Several key factors, including ionic radius, hydration energy, electronegativity, charge density, and the nature of functional groups on the adsorbent surface, play a crucial role in determining the adsorption affinity of metal ions.^4,10,12,33,39–43^ These factors collectively influence the stability and strength of interactions between metal ions and the GA-NiMMPs, ultimately dictating the preferential adsorption of Cd(ii).

One significant parameter contributing to this selectivity is the hydrated ionic radius. Cd(ii) has a larger ionic radius (0.426 nm) compared to Cu(ii) (0.295 nm),^4,39^ which facilitates stronger and more effective coordination with the functional groups present on the GA-NiMMPs. This larger ionic radius allows Cd(ii) to form more stable interactions with carboxyl (–COO^−^), and hydroxyl (–OH) groups, which have been confirmed through FTIR analysis (Fig. 1-II). Moreover, Cd(ii) generally exhibits a higher coordination number (typically 6) than Cu(ii), which often forms square-planar or distorted octahedral complexes with a coordination number of 4–6.^4,39^ The ability of Cd(ii) to engage with a greater number of binding sites on the adsorbent further enhances its adsorption capacity. Hydration energy plays a critical role in the adsorption process by determining how easily metal ions shed their hydration shells to interact with the adsorbent surface. Cd(ii) has a lower hydration energy (−1807 kJ mol^−1^) than Cu(ii) (−2100 kJ mol^−1^), meaning that Cd(ii) ions are less strongly solvated in aqueous solutions.^33,41,42^ The weaker solvation of Cd(ii) allows for easier dehydration and subsequent interaction with the adsorbent's active sites. In contrast, the higher hydration energy of Cu(ii) results in stronger solvation, making it less available for adsorption and limiting its ability to effectively bind with the functional groups on GA-NiMMPs.

Electronegativity and charge density also influence adsorption behavior. Cd(ii) has a lower electronegativity (1.69 on the Pauling scale) than Cu(ii) (1.90), which contributes to reduced electrostatic repulsion between Cd(ii) ions and the electron-rich functional groups on the GA-NiMMPs surface.^4,21,33,42^ This lower repulsion allows Cd(ii) to establish stronger and more stable complexes with the adsorbent. Additionally, Cd(ii) possesses a lower charge density (59*C* mm^−3^) than Cu(ii) (71*C* mm^−3^), further reducing electrostatic repulsion and promoting adsorption. The relatively lower charge density of Cd(ii) facilitates stronger binding with the adsorbent's functional groups, whereas the higher charge density of Cu(ii) results in stronger electrostatic interactions with surrounding water molecules, decreasing its adsorption efficiency.^43^ From a coordination chemistry perspective, the Hard-Soft Acid-Base (HSAB) theory provides further insight into the preferential adsorption of Cd(ii). Cadmium(ii) is classified as a softer Lewis acid, whereas Cu(ii) is a borderline Lewis acid.^33,44^ The functional groups present on GA-NiMMPs, such as hydroxyl (–OH), and carboxyl (–COO^−^), behave as intermediate to soft bases. As a result, Cd(ii), being a softer acid, exhibits a stronger affinity for these functional groups due to more favorable orbital overlap and bonding interactions. In contrast, Cu(ii), with its borderline acid character, does not interact as effectively with these groups, leading to reduced adsorption.

The structural and chemical properties of GA-NiMMPs further enhance the selective adsorption of Cd(ii). The presence of multiple active functional groups provides diverse binding sites, enabling complexation, electrostatic attraction, and hydrogen bonding with metal ions. The larger ionic radius and lower hydration energy of Cd(ii) allow it to interact more effectively with these functional groups, forming stable complexes that improve adsorption efficiency. On the other hand, Cu(ii), with its smaller ionic radius and higher hydration energy, encounters steric and solvation-related challenges that hinder its ability to form similarly stable interactions.

Overall, the superior adsorption of Cd(ii) onto GA-NiMMPs can be attributed to its larger ionic radius, lower hydration energy, reduced charge density, and favorable interactions with functional groups based on HSAB theory. These factors collectively enhance the selectivity of GA-NiMMPs for Cd(ii) over Cu(ii), making it an effective adsorbent for cadmium removal in environmental remediation applications. This preferential adsorption of Cd(ii) over Cu(ii) from aqueous solutions has also been observed with other sorbents, including Ag-MOF/chitosan composite sponges,^12^ Fly Ash-Based Linde F(K) Zeolite,^13^ exhausted copper slag-supported sulfidized nanoscale zerovalent iron,^14^ dithiocarbamate-functionalized activated carbon,^15^*Cystoseira sedoide* algae,^16^ and ethylenediamine-functionalized chelating resin.^28^ These findings provide valuable insights for optimizing adsorbents for selective heavy metal removal, particularly in wastewater treatment processes where cadmium contamination is a concern.

The comparative evaluation of various sorbents for Cu(ii) and Cd(ii) removal from aqueous solutions provides valuable insights into the performance of the newly synthesized GA-MMPs and GA-NiMMPs. As shown in Table 6, the adsorption data exhibit clear trends in removal efficiencies, highlighting the enhanced performance of GA-NiMMPs relative to both GA-MMPs and several benchmark materials reported in the literature. For Cu(ii) adsorption, GA-NiMMPs achieved a capacity of 27.7 mg g^−1^, surpassing conventional sorbents such as biochar derivatives (12.2–14.7 mg g^−1^),^10^ natural biomass-based materials like pata-de-vaca leaves (15.1 mg g^−1^),^38^ and several zeolite-based adsorbents.^13,39^ In the case of Cd(ii), GA-NiMMPs reached 30.4 mg g^−1^, outperforming engineered materials including CuO-modified ceramic membranes (12.4 mg g^−1^)^4^ and Zn_0_._6_Mn_0_._4_Fe_2_O_4_/PUF nanoparticles (11.1 mg g^−1^).^18^

**Table 6:** Comparison of sorption capacities of various sorbents for Cd(ii) and Cu(ii) removal from aqueous solutions

Sorbent type	*C*_o_, mg L^−1^	Temp.	pH	Time, min	*q*_e_, mg g^−1^		*R*
					Cu(ii)	Cd(ii)	
CuO-modified ceramic membrane	6.3–44.5	25	4.0	120	13.04	12.4	4
Willow wood biochar (WWB)	0.05–160	25	5.2	1440	12.2	35.2	10
Cattle manure biochar (CMB)					14.7	31.3	
Chitosan-vermiculite composite	—	25	8.0	360	116.2	147.6	11
Ag-MOF/CSC composite sponge	—	25	5.0	60	94.7	193.3	12
Fly ash-based Linde F(K) zeolite	—	25	6.0	480	18.5	21.6	13
Sulfidized nanoscale zero-valent iron	50–400	25	6.0	2880	85.1	126.9	14
*Cystoseira sedoide* alga	25–150	25	5.0	180	14.66	23.78	16
Magnetite–chitosan composite	0–200	25	5.0	10	26.28	18.67	17
Zn_0.6_Mn_0.4_Fe_2_O_4_/PUF nanocomposite	0.2–300	25	6.5	60	20.4	11.1	18
Magnetic iron-modified calcium silicate hydrate	—	25	4.0	300	26.12	16.39	19
Pectin hydrogel/Fe_3_O_4_/bentonite	100–300	25	4.7	45	36.8	35.5	36
Pata-de-vaca leaves	1–400	25	5.0	240	15.1	12.7	38
NaP1 zeolite	0–223.5	22	5.6	180	20.9	26.9	39
Nanosized titanate composites (Na_*x*_Fe_*y*_MgiTiO_*z*_)	300–450	20	—	420	13.8	8.8	45
Commercial active carbon (Purolite AC 20)	50–200	25	5.0	360	8.47	12.6	46
Biochar					24.9	33.9	
GA-NiMMPs	20–200	25	6.0	240	27.7	30.4	PW

The superior performance of GA-NiMMPs can be attributed to several synergistic factors. The incorporation of nickel likely introduces additional active sites, enhancing metal ion binding capacity. Moreover, the combination of gum Arabic—a natural polymer rich in functional groups—with the magnetite–nickel matrix may facilitate multiple adsorption mechanisms, including electrostatic attraction, surface complexation, and ion exchange. The mesoporous architecture further contributes by offering a high surface area, thereby increasing the number of accessible binding sites. It is also noteworthy that both GA-based sorbents exhibited higher affinity for Cd(ii) over Cu(ii), a trend consistent with other reported adsorbents. This selectivity may be influenced by intrinsic ionic properties such as hydration energy, ionic radius, and electronegativity differences between the two metal ions.

The experimental conditions employed (pH 6.0, 25 °C, and a contact time of 240 minutes) were carefully selected to simulate practical application scenarios, offering a balance between adsorption efficiency and operational feasibility. Additionally, the initial metal concentration range (20–200 mg L^−1^) reflects typical contamination levels found in industrial effluents. In conclusion, the newly developed GA-NiMMPs exhibit promising potential for the remediation of heavy metal-contaminated water, particularly for the removal of Cd(ii) and Cu(ii). Their adsorption capacities compare favorably with many established sorbents, positioning them as effective, eco-friendly materials for future water treatment technologies.

### 3.5. Thermodynamic investigation

**Fig. 7:**
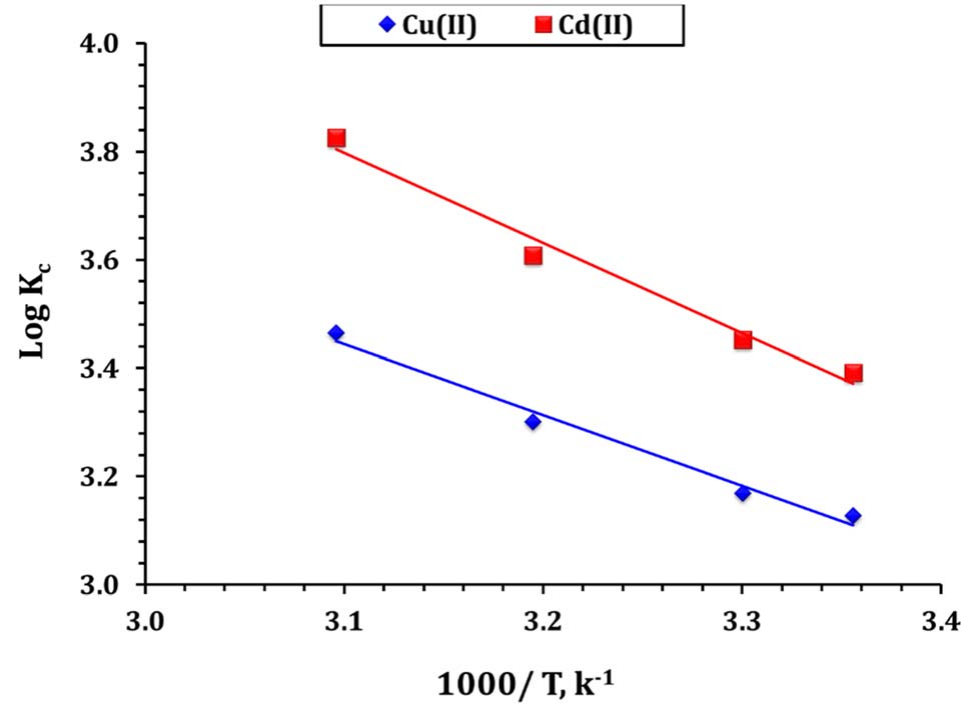
Thermodynamic profile for the capture process (initial concentration of 60 ppm; pH 5.9; ligand dose of 4.0 g L^−1^; shaking time is 240 min).

The thermodynamic investigation of Cd(ii) and Cu(ii) adsorption onto GA-NiMMPs provides crucial insights into the spontaneity, feasibility, and energetic nature of the adsorption process.^34,35^ The thermodynamic parameters of adsorption (Fig. 7 and Table 7) were calculated using standard Gibbs free energy (Δ*G*°), enthalpy (Δ*H*°), and entropy (Δ*S*°) equations across temperatures from 25–50 °C. The negative Δ*G*° values confirmed the spontaneous nature of Cd(ii) and Cu(ii) adsorption, with increasing magnitude at higher temperatures (*e.g.*, from −17.8 to −21.4 kJ mol^−1^ for Cu(ii), and −19.3 to −23.6 kJ mol^−1^ for Cd(ii)), reflecting enhanced spontaneity with thermal energy.^11,12^ The endothermic nature of the adsorption is confirmed by the positive enthalpy values (Δ*H* = 31.88 kJ mol^−1^ for Cd(ii) and 25.07 kJ mol^−1^ for Cu(ii)).^14–16^ The Δ*H*° value being less than 84 kJ mol^−1^ suggests that the sorption process primarily occurs through a physical mechanism (physisorption).^34,35^ These results, coupled with Temkin model findings, suggest a dominant physisorption process complemented by chemical binding.

**Table 7:** The values of the thermodynamic parameters

	Δ*G* (kJ mol^−1^)				Δ*H*	Δ*S*
	20 °C	30 °C	40 °C	50 °C	(kJ mol^−1^)	(J mol^−1^ K^−1^)
Cu(ii)	−17.8	−18.3	−19.7	−21.4	25.0	144.0
Cd(ii)	−19.3	−20.0	−21.6	−23.6	31.8	171.9

The entropy change (Δ*S*° = 144.03 J mol^−1^ K^−1^ for Cu(ii), 171.94 J mol^−1^ K^−1^ for Cd(ii)) suggests increased randomness at the solid–solution interface, attributed to water molecule displacement and enhanced molecular freedom upon metal binding.^14–16^ The higher Δ*S* for Cd(ii) compared to Cu(ii) suggests a more significant structural reorganization upon adsorption, which is consistent with its higher affinity for GA-NiMMPs. Research on the adsorption of cadmium(ii), and copper(ii) ions onto various materials—including chitosan–vermiculite composite,^11^ Ag-MOF/chitosan composite sponges,^12^ exhausted copper slag-supported sulfidized nanoscale zerovalent iron,^14^ dithiocarbamate-functionalized activated carbon,^15^*Cystoseira sedoide* algae,^16^ and ethylenediamine-functionalized chelating resin^28^ has shown the process to be spontaneous, thermodynamically favorable, and endothermic.

Overall, the thermodynamic evaluation highlights the fundamental energetic feasibility and thermal responsiveness of the adsorption process. The negative Δ*G*° values across the tested temperature range confirm that the adsorption of Cd(ii) and Cu(ii) onto GA-NiMNPs occurs spontaneously, a desirable attribute for practical wastewater treatment systems. The positive enthalpy values indicate that the process is endothermic, gaining in efficiency with increasing temperature, which is particularly beneficial for real-world applications involving warm industrial effluents. Meanwhile, the positive entropy changes reflect increased molecular disorder at the interface, attributed to the release of water molecules and enhanced freedom of adsorbed ions upon complexation. These thermodynamic parameters reinforce the findings of kinetic and isotherm studies, suggesting a mixed-mode adsorption involving both physical and chemical interactions. The compatibility of GA-NiMNPs with varying thermal conditions, along with their high binding affinity and adaptable surface chemistry, underscores their robustness and versatility as a sustainable adsorbent in dynamic environmental systems.

### 3.6. Proposed adsorption mechanisms

Understanding adsorption mechanisms for heavy metal removal is essential for rational adsorbent design and process optimization. This knowledge enables targeted modifications to enhance selectivity, capacity, and regenerability while minimizing resource consumption. The effectiveness of composites in absorbing metal ions is influenced by various factors including the chemistry of the metal ion in solution, and the morphology and texture properties of the composites.^4,28,46^ The adsorption of metal ions using composites is a complex process involving several mechanisms, including physical adsorption, electrostatic interaction, ion exchange, precipitation, and complexation.^10,12,14,15,18,19^

The removal of Cu(ii) and Cd(ii) from aqueous solutions proceeds through a hierarchical sequence of mechanistic pathways, beginning with surface interactions and progressing to specific chemical binding processes. Initially, electrostatic attraction serves as the primary driving force, drawing positively charged metal ions from bulk solution toward negatively charged adsorbent surfaces.^15,18^ This physical adsorption phase is particularly influenced by solution pH, which determines both the surface charge of the adsorbent (point of zero charge) and the speciation of metal ions.^10^ Following surface contact, multiple chemical binding mechanisms operate simultaneously with varying contributions. Coordination chemistry dominates through oxygen-containing functional groups—carboxyl (–COOH), hydroxyl (–OH), and phenolic (R-OH)—which undergo deprotonation to form stable metal complexes such as –(COO)_2_M, –COOM^+^, –(O)_2_M, and –OM^+^.^46^ Importantly, lattice Ni–O and Fe–O sites within the GA–NiMMPs framework contribute significantly to surface complexation, providing additional coordination centers for Cd(ii) and Cu(ii). Nitrogen-containing groups (–NH_2_) demonstrate effective coordination with both metals, while interestingly, hydroxyl groups show preferential binding to Cd(ii) over Cu(ii).^28^ Ion exchange represents another significant mechanism, particularly in silicate-based adsorbents where structural cations (often Ca^2+^) are displaced by incoming Cu(ii) and Cd(ii) ions.^19^ In more advanced materials, surface complexation and chelation create highly stable metal–ligand structures,^15^ while π–π interactions with aromatic structures in carbonaceous materials offer additional binding sites.^12^ Material-specific mechanisms include precipitation processes, such as the formation of metal hydroxides catalyzed by silanol groups in silicates,^19^ and redox reactions, exemplified by the reduction of Cu(ii) to Cu^0^ on sulfurized nanoscale zero-valent iron surfaces.^14^ The synergistic integration of these mechanisms—electrostatic attraction, coordination bonding, ion exchange, precipitation, and redox transformations—varies with adsorbent composition and target metal, explaining the observed differences in removal efficiencies. This mechanistic understanding highlights the importance of rational adsorbent design that strategically incorporates functional groups with optimal affinity and selectivity for Cu(ii) and Cd(ii),^4^ enabling the development of more effective water purification technologies.

The adsorption of Cd(ii) and Cu(ii) ions onto GA-NiMMPs is a multifaceted process driven by the material's unique structural and functional properties, as revealed by comprehensive characterization and adsorption studies. The mesoporous structure and high surface area (29.32 m^2^ g^−1^) of GA-NiMMPs, confirmed by BET and SEM analyses (Table 2 and Fig. 2-I respectively), provide an extensive network of accessible active sites, facilitating efficient metal ion binding. The presence of functional groups such as hydroxyl (–OH), and carboxyl (–COO^−^), identified through FTIR analysis (Fig. 1-II), plays a pivotal role in enhancing the material's adsorption capacity through multiple mechanisms, including coordination bonding, electrostatic attraction, hydrogen bonding, and surface complexation. These functional groups act as electron donors, forming strong coordination bonds with Cd(ii) and Cu(ii) ions, which act as Lewis acids. The carboxyl groups, in particular, deprotonate at moderate pH values (pH ∼6.0) to form carboxylate anions (–COO^−^), enabling chelation and electrostatic interactions with the metal ions. The negative zeta potential of −21.32 mV (Table 1) further supports the electrostatic attraction mechanism, as the negatively charged adsorbent surface attracts positively charged Cd^2+^ and Cu^2+^ ions, enhancing adsorption efficiency.

Kinetic studies reveal that the adsorption process is dominated by chemisorption, as evidenced by the excellent fit of the pseudo-second-order model, which indicates that chemical interactions are the rate-limiting step. The initial rapid adsorption phase is attributed to the high density of active sites on the adsorbent surface, while the slower phase involves intraparticle diffusion, as confirmed by the Weber–Morris model. The mesoporous structure of GA-NiMMPs, with an average pore size of 688.3 nm (Table 1), facilitates efficient diffusion of metal ions to the interior active sites, ensuring rapid and effective adsorption. The Elovich model further supports the heterogeneous nature of the adsorbent surface, highlighting the role of multiple binding sites in the adsorption process.

Isotherm studies demonstrate that the adsorption of Cd(ii) and Cu(ii) follows a monolayer coverage mechanism, as indicated by the excellent fit of the Langmuir model. The maximum adsorption capacities (*q*_m_) of 30.4 mg g^−1^ for Cd(ii) and 27.7 mg g^−1^ for Cu(ii) (Table 5) reflect the material's high affinity for both metal ions. The Freundlich and Temkin models further underscore the heterogeneous nature of the adsorbent surface and the role of adsorbate–adsorbent interactions in enhancing adsorption performance. The Sips model, which combines features of the Langmuir and Freundlich models, confirmed that the adsorption process is predominantly monolayer in nature, consistent with the homogeneous distribution of functional groups on the adsorbent surface. Thermodynamic investigations reveal that the adsorption process is spontaneous, endothermic, and entropy-driven, as evidenced by the negative Gibbs free energy change (Δ*G*), positive enthalpy change (Δ*H*), and positive entropy change (Δ*S*) (Table 7). The occurrence of physisorption during the elimination of cadmium(ii) and copper(ii) is also confirmed by thermodynamic and Temkin isotherm analyses, which indicate Δ*H*° values of 25.0 and 31.8 kJ mol^−1^ (Table 7) and heats of adsorption of 414.3 and 428.9 (Table 5) for copper and cadmium ions respectively. These findings suggest that physisorption, *via* weak van der Waals interactions, plays a role in the removal of metal cations.^14,15^

Structural characterization by EDX (Fig. 1-I), and XRD (Fig. 1-III) further supports the adsorption mechanism, revealing the successful incorporation of Ni within the magnetite framework, which enhances the density of active sites and contributes to improved metal binding. The morphological features observed in SEM images indicate a rough and porous surface that maximizes contact with metal ions, optimizing the adsorption process. The thermodynamic findings confirm that adsorption is spontaneous, with increasingly negative Gibbs free energy (Δ*G*) values at higher temperatures, further validating the favorability of the process. The superior adsorption of Cd(ii) compared to Cu(ii) is attributed to its more effective interaction with GA-NiMMPs due to its softer Lewis acid nature, allowing for stronger chelation with the functional groups present on the sorbent.

**Fig. 8:**
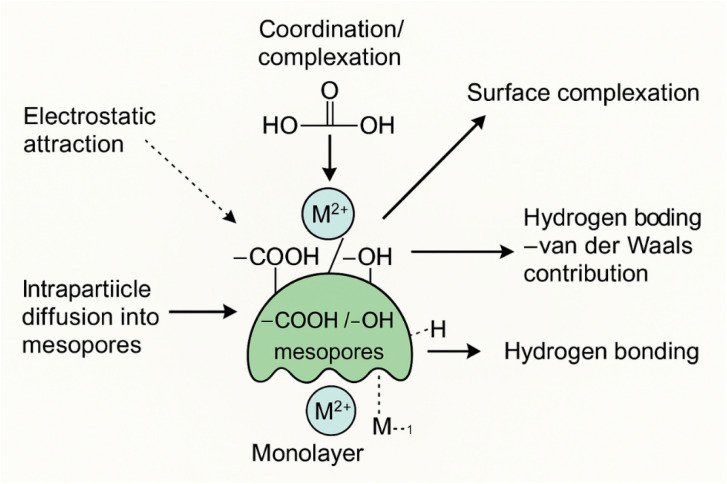
Schematic representation of the main proposed adsorption mechanisms for Cd(ii) and Cu(ii) onto GA-NiMMPs.

Overall, the adsorption of Cd(ii) and Cu(ii) onto GA-NiMMPs is a complex but highly efficient process, integrating multiple interaction mechanisms-electrostatic attraction, coordination/complexation (*via* GA and Ni/Fe–O sites), ion exchange, hydrogen bonding, and van der Waals interactions-that are strongly supported by kinetic, isotherm, thermodynamic, and structural characterization insights. This comprehensive understanding of the adsorption behavior highlights the potential of GA-NiMMPs as a highly effective and selective material for heavy metal remediation in environmental applications. The main interaction pathways are summarized in Fig. 8, illustrating electrostatic attraction, coordination/complexation, ion exchange, hydrogen bonding, and physical adsorption (van der Waals), which act synergistically to capture Cd(ii) and Cu(ii) ions.

### 3.7. Desorption investigation and application in waste raffinate solution (case study)

The desorption investigation provides valuable insights into the regeneration potential of GA-NiMMPs and the effectiveness of different eluents in recovering Cd(ii) and Cu(ii) from the sorbent. Efficient desorption is essential for maintaining the reusability of the material and reducing operational costs in wastewater treatment applications. The study evaluated desorption efficiency using sulfuric (H_2_SO_4_), hydrochloric (HCl), and nitric (HNO_3_) acids at a concentration of 1.0 M, revealing notable differences in their ability to release the adsorbed metal ions. As shown in Fig. S2, H_2_SO_4_ demonstrated the highest desorption efficiency, reaching 93.1% for Cd(ii) and 92.1% for Cu(ii), followed by HNO_3_ with efficiencies of 86.9% for Cd(ii) and 66.9% for Cu(ii). HCl exhibited the lowest desorption performance, achieving 82.3% for Cd(ii) and only 46.3% for Cu(ii), suggesting that the strength of metal–sorbent interactions influences the ease of desorption. These findings confirm sulfuric acid as the most effective desorbing agent, making it an optimal choice for regenerating GA-NiMMPs while preserving its adsorption capacity for reuse. The ability to achieve high desorption efficiencies under controlled conditions enhances the practical applicability of GA-NiMMPs in heavy metal remediation, reinforcing its potential as a cost-effective and sustainable solution for wastewater treatment.

To evaluate the real-world applicability of GA-NiMNPs beyond controlled laboratory environments, the material was tested on a chemically complex waste raffinate solution containing a mixture of heavy metals, and alkali metals. The sample was sourced from the Central Analytical Laboratory of the Nuclear Materials Authority (NMA), Cairo, Egypt, where multi-element chemical analyses and metal dissolution procedures generate composite aqueous effluents. The tested solution included toxic and trace-level metals such as Cd(ii), Cu(ii), As(iii/v), Co(ii), Ni(ii), Cr(iii), and Fe(iii), as well as background electrolytes such as Na^+^ and K^+^, and trace amounts of organic dyes (*e.g.*, methylene blue) used in analytical quality control. Initial concentrations of the target contaminants ranged from 6.0 to 65.8 mg L^−1^ (see Fig. S3).

Adsorption experiments were conducted under previously optimized conditions: GA-NiMNPs dosage of 4.0 g L^−1^, solution pH ∼6.0, contact time of 240 minutes, and ambient temperature. Following treatment, the removal efficiencies (*E*%) of the major contaminants were assessed and visually summarized in Fig. S3. GA-NiMNPs exhibited the highest removal for Cd(ii) and Cu(ii), achieving efficiencies of 40.1% and 25.3%, respectively—demonstrating strong affinity toward these priority pollutants and consistent with batch and isotherm findings. Other metal ions, including Co(ii), Ni(ii), Cr(iii), and Fe(iii), showed moderate removal (15–23%), which can be attributed to electrostatic interactions and coordination with carboxyl and hydroxyl groups on the gum Arabic-functionalized surface. Arsenic (present as As(iii/v)) exhibited limited uptake (∼10%), likely due to the weak affinity of its oxyanionic forms under near-neutral pH. Common alkali metals Na^+^ and K^+^ were minimally removed (<5%) due to their low charge density and limited interaction potential.

Although adsorption performance in this multicomponent matrix was lower than in synthetic single-ion systems, GA-NiMNPs maintained structural integrity and functional activity under highly competitive conditions. The moderate reduction in performance is attributed to matrix interference effects. Specifically, the presence of organic dyes such as methylene blue may interfere by forming weak metal-dye complexes that hinder the availability of free Cu^2+^ and Cd^2+^ ions. Additionally, the presence of competing cations (*e.g.*, Cr^3+^, Fe^3+^, Ni^2+^) and the high ionic strength of the solution may cause electrostatic shielding and partial occupation of available adsorption sites, thereby reducing overall uptake. Nevertheless, the sorbent preserved a consistent preferential binding for Cd(ii) over Cu(ii), aligning with earlier observations and affinity trends based on ion size, hydration energy, and softness.

These results underscore two key advantages of GA-NiMNPs: (1) their chemical robustness in diverse and ion-rich matrices, and (2) their maintained selectivity in the presence of multiple interfering species. From a practical standpoint, the ability to capture cadmium and copper from real, multi-element wastewaters highlights the material's value for sustainable water treatment applications. This case study supports the broader applicability of GA-NiMNPs in environmental remediation settings and affirms their role in circular economy strategies for biomass-derived sorbents.

## 4. Conclusion

This study demonstrates the successful green synthesis of GA-NiMMPs using gum Arabic as a natural stabilizer and surface modifier. The composite was designed to remove toxic divalent metal ions from aqueous systems, including real industrial wastewater. Structural and surface characterizations confirmed the formation of a mesoporous, magnetically separable, and functionally active material, with effective incorporation of GA and Ni onto the magnetite matrix as evidenced by FTIR, EDX, and XRD analyses. The role of nickel was found to enhance surface reactivity and promote additional coordination sites, particularly favoring Cd(ii) uptake under binary conditions. Batch adsorption experiments revealed high removal efficiencies for both Cd(ii) and Cu(ii), with a stronger adsorption preference for Cd(ii). Kinetic and isotherm modeling confirmed pseudo-second-order kinetics and monolayer chemisorption behavior, consistent with selective and energetically favorable interactions. The material's performance remained stable in real industrial effluent containing multiple heavy metals and organic residues, supporting its robustness and applicability under complex environmental conditions. The use of a biodegradable biopolymer, eco-friendly processing, and magnetically retrievable particles align this work with circular economy principles and green chemistry strategies. In summary, this study contributes to the development of sustainable bio-derived adsorbents for water purification by presenting a scalable and selective material for heavy metal remediation. The insights gained support the rational design of multifunctional sorbents for real-world water treatment applications and align with the scope of *RSC Advances* at the intersection of chemistry, sustainability, and applied innovation.

## Conflicts of interest

The authors of this article would like to confirm that all of them have no conflict of interests with any organization or any person and the funding body is listed.

## Supplementary Material

RA-015-D5RA04152J-s001

## Data Availability

The datasets used and/or analyzed during the current study are available from the corresponding author on reasonable request. The SI file includes adsorption kinetic and isotherm model equations with corresponding fitting parameters, detailed thermodynamic calculations, and supporting figures and tables (Fig. S1–S3 and Tables S1 and S2) that complement and extend the results presented in the main article. See DOI: https://doi.org/10.1039/d5ra04152j.

## References

[cit1] Tiwari A. K., Pal S. L., Srivastava N., Shah M., Ahmad I., Alshahrani M. Y., Pal D. B. (2023). Bioadsorbent and adsorbent-based heavy metal removal technologies from wastewater: new insight. Biomass Convers. Biorefin..

[cit2] Sireesha S., Upadhyay U., Sreedhar I. (2022). Comparative studies of heavy metal removal from aqueous solution using novel biomass and biochar-based adsorbents: characterization, process optimization, and regeneration. Biomass Convers. Biorefin..

[cit3] Saravanan P., Saravanan V., Rajeshkannan R., Arnica G., Rajasimman M., Gurunathan B., Pugazhendhi A. (2024). Comprehensive review on toxic heavy metals in the aquatic system: sources, identification, treatment strategies, and health risk assessment. Environ. Res..

[cit4] Mahatmanti F. W., Jumaeri J., Kusumastuti E. (2023). The adsorption behavior of individual Cu(II), Zn(II), and Cd(II) ions over a CuO-modified ceramic membrane synthesized from fly ash. J. King Saud Univ. Sci..

[cit5] Budi H. S., Opulencia M. J. C., Afra A., Abdelbasset W. K., Abdullaev D., Majdi A., Taherian M., Ekrami H. A., Mohammadi M. J. (2024). Source, toxicity and carcinogenic health risk assessment of heavy metals. Rev. Environ. Health.

[cit6] Benalia M. C., Youcef L., Bouaziz M. G., Achour S., Menasra H. (2022). Removal of heavy metals from industrial wastewater by chemical precipitation: mechanisms and sludge characterization. Arabian J. Sci. Eng..

[cit7] Fu Z. J., Jiang S. K., Chao X. Y., Zhang C. X., Shi Q., Wang Z. Y., Liu M. L., Sun S. P. (2022). Removing miscellaneous heavy metals by all-in-one ion exchange-nanofiltration membrane. Water Res..

[cit8] Jayaraman J., Kumaraswamy J., Rao Y. K., Karthick M., Baskar S., Anish M., Sharma A., Yadav A. S., Alam T., Ammarullah M. I. (2024). Wastewater treatment by algae-based membrane bioreactors: a review of the arrangement of a membrane reactor, physico-chemical properties, advantages and challenges. RSC Adv..

[cit9] Gunarathne V., Rajapaksha A. U., Vithanage M., Alessi D. S., Selvasembian R., Naushad M., You S., Oleszczuk P., Ok Y. S. (2022). Hydrometallurgical processes for heavy metals recovery from industrial sludges. Crit. Rev. Environ. Sci. Technol..

[cit10] Wang S., Kwak J. H., Islam M. S., Naeth M. A., El-Din M. G., Chang S. X. (2020). Biochar surface complexation and Ni(II), Cu(II), and Cd(II) adsorption in aqueous solutions depend on feedstock type. Sci. Total Environ..

[cit11] Salih S. S., Shihab M. A., Mohammed H. N., Kadhom M., Albayati N., Ghosh T. K. (2024). Chitosan-vermiculite composite adsorbent: Preparation, characterization, and competitive adsorption of Cu(II) and Cd(II) ions. J. Water Process Eng..

[cit12] Nassef H. M., Al-Hazmi G. A. A., Alayyafi A. A., El-Desouky M. G., El-Bindary A. A. (2024). Synthesis and characterization of new composite sponge combining of metal-organic framework and chitosan for the elimination of Pb(II), Cu(II) and Cd(II) ions from aqueous solutions: batch adsorption and optimization using Box-Behnken design. J. Mol. Liq..

[cit13] Cheng T., Chen C., Tang R., Han C. H., Tian Y. (2018). Competitive adsorption of Cu, Ni, Pb, and Cd from aqueous solution onto fly ash-based Linde F(K) Zeolite. Iran. J. Chem. Chem. Eng..

[cit14] Shi L., Deng Q., Guo L., Du Y., Du D., Zhang T. C. (2023). Efficient removal of Cd(II), Cu(II), and
Pb(II) in aqueous solutions by exhausted copper slag supported sulfidized nanoscale zerovalent iron. Sep. Purif. Technol..

[cit15] Niu H. Y., Li X., Li J. (2022). Dithiocarbamate modification of activated carbon for the efficient removal of Pb(II), Cd(II), and Cu(II) from wastewater. New J. Chem..

[cit16] Bengourna N., Belguidoum K., Khalla D., Nacef M., Kouadri I., Benhamida A., Amira-Guebailia H., Brouk A. E., Affoune A. M., Satha H. (2024). Exploring the efficacy of Cystoseira sedoide alga for cadmium and copper biosorption: an integrated experimental and computational study. RSC Adv..

[cit17] Hu C., Zheng Z., Huang M., Yang F., Wu X., Zhang A. (2023). Adsorption characterization of Cu(II) and Cd(II) by a magnetite–chitosan composite: kinetic, thermodynamic and equilibrium studies. Polymers.

[cit18] Azeem S. M. A., Wahsh M. M., Youssef F. H., Ibrahim A. M., Burham N. (2022). Magnetic nanocomposite of zinc–manganese ferrite/polyurethane foam for adsorption of copper and cadmium from water. Desalin. Water Treat..

[cit19] Valenzuela F., Quintana G., Briso A., Ide V., Basualto C., Gaete J., Montes G. (2021). Cu(II), Cd(II), Pb(II) and As(V) adsorption from aqueous solutions using magnetic iron-modified calcium silicate hydrate: adsorption kinetic analysis. J. Water Process Eng..

[cit20] Mustafa H. J., Al-Saadi T. M. (2021). Effects of gum arabic-coated magnetite nanoparticles on the removal of Pb ions from aqueous solutions. Iraqi J. Sci..

[cit21] Nasef S., ElNesr E., Hafez F., Badawy N., Slim S. (2020). Gamma irradiation induced preparation of gum arabic/poly(vinyl alcohol) copolymer hydrogels for removal of heavy metal ions from wastewater. Arab. J. Nucl. Sci. Appl..

[cit22] Birniwa A. H., Mohammad R. E. A., Ali M., Rehman M. F., Abdullahi S. S. A., Eldin S. M., Mamman S., Sadiq A. C., Jagaba A. H. (2022). Synthesis of gum arabic magnetic nanoparticles for adsorptive removal of ciprofloxacin: equilibrium, kinetic, thermodynamics studies, and optimization by response surface methodology. Separations.

[cit23] Ali I. H., Bani-Fwaz M. Z., El-Zahhar A. A., Marzouki R., Jemmali M., Ebraheem S. M. (2021). Gum arabic-magnetite nanocomposite as an eco-friendly adsorbent for removal of lead(II) ions from aqueous solutions: equilibrium, kinetic and thermodynamic studies. Separations.

[cit24] Elwakeel K. Z., Ahmed M. M., Akhdhar A., Alghamdi H. M., Sulaiman M. G., Hamza M. F., Khan Z. A. (2023). Effect of the magnetic core in alginate/gum composite on adsorption of divalent copper, cadmium, and lead ions in the aqueous system. Int. J. Biol. Macromol..

[cit25] Fablet L., Pédrot M., Choueikani F., Kieffer I., Proux O., Cagnart V., Yomogida T., Marsac R. (2025). Nickel binding with magnetite nanoparticles. Environ. Sci. Nano.

[cit26] Lemine O. M., Ghiloufi I., Bououdina M., Khezami L., M’hamed M. O., Hassan A. T. (2014). Nanocrystalline Ni doped α-Fe2O3 for adsorption of metals from aqueous solution. J. Alloys Compd..

[cit27] El Malti W., Hijazi A., Abou Khalil Z., Yaghi Z., Medlej M. K., Reda M. (2022). Comparative study of the elimination of copper, cadmium, and methylene blue from water by adsorption on the citrus Sinensis peel and its activated carbon. RSC Adv..

[cit28] Chen Y., Jia S., Zhao W., Song J., Li Y., Wang H. (2022). Ethylenediamine functionalized chelating resin for removal of Cu(II) and Cd(II) from aqueous solution. Desalin. Water Treat..

[cit29] Hu Q., Pang S., Wang D. (2022). In-depth insights into mathematical characteristics, selection criteria and common mistakes of adsorption kinetic models: a critical review. Sep. Purif. Rev..

[cit30] González-López M. E., Laureano-Anzaldo C. M., Pérez-Fonseca A. A., Arellano M., Robledo-Ortíz J. R. (2022). A critical overview of adsorption models linearization: methodological and statistical inconsistencies. Sep. Purif. Rev..

[cit31] Chen X., Hossain M. F., Duan C., Lu J., Tsang Y. F., Islam M. S., Zhou Y. (2022). Isotherm models for adsorption of heavy metals from water—a review. Chemosphere.

[cit32] Majd M. M., Kordzadeh-Kermani V., Ghalandari V., Askari A., Sillanpää M. (2022). Adsorption isotherm models: a comprehensive and systematic review (2010–2020). Sci. Total Environ..

[cit33] Taha M. H. (2021). Sorption of U(VI), Mn(II), Cu(II), Zn(II), and Cd(II) from multi-component phosphoric acid solutions using MARATHON C resin. Environ. Sci. Pollut. Res..

[cit34] Masoud A. M., Ammar H., Elzoghby A. A., El Agamy H. H., Taha M. H. (2025). Rare earth elements adsorption from phosphoric acid solution using dendrimer modified silica gel as well as kinetic, isotherm, and thermodynamic studies. J. Rare Earths.

[cit35] Ebelegi A. N., Ayawei N., Wankasi D. (2020). Interpretation of adsorption thermodynamics and kinetics. Open J. Phys. Chem..

[cit36] Maleki S. T., Beigi P., Babamoradi M. (2023). Synthesis of pectin hydrogel/Fe3O4/Bentonite and its use for the adsorption of Pb(II), Cu(II), and Cd(II) heavy metals from aqueous solutions. Mater. Sci. Eng., B.

[cit37] Masoud A. M. (2025). Cobalt and hydroxy magnetite doped Arabic gum as mesoporous composite for efficient removal of uranium (VI) from aqueous solution. Environ. Technol..

[cit38] Jorgetto A. D. O., da Silva A. C., Wondracek M. H., Silva R. I., Velini E. D., Saeki M. J., Pedrosa V. A., Castro G. R. (2015). Multilayer adsorption of Cu(II) and Cd(II) over Brazilian Orchid Tree (Pata-de-vaca) and its adsorptive properties. Appl. Surf. Sci..

[cit39] Visa M. (2016). Synthesis and characterization of new zeolite materials obtained from fly ash for heavy metals removal in advanced wastewater treatment. Powder Technol..

[cit40] Peng H., Gao P., Chu G., Pan B., Peng J., Xing B. (2017). Enhanced adsorption of Cu(II) and Cd(II) by phosphoric acid-modified biochars. Environ. Pollut..

[cit41] Chu Z., Gu W., Li Y. (2018). Adsorption mechanism of heavy metals in heavy metal/pesticide coexisting sediment systems through fractional factorial design assisted by 2D-QSAR models. Pol. J. Environ. Stud..

[cit42] Fan X., Liu H., Anang E., Ren D. (2021). Effects of electronegativity and hydration energy on the selective adsorption of heavy metal ions by synthetic NaX zeolite. Materials.

[cit43] Noun F., Jury E. A., Naccache R. (2021). Elucidating the quenching mechanism in carbon dot-metal interactions—designing sensitive and selective optical probes. Sensors.

[cit44] Xia Y., Liao G., Wang Z., Wei Y., Liu H., Tang Y., Tang H., Liu X., Shi J., Liu C. (2025). Efficient and deep adsorption of thallium(I) from complex water based on hard-soft acid-base theory. Sep. Purif. Technol..

[cit45] Wang A., Si Y., Yin H., Chen J., Huo J. (2019). Synthesis of Na-, Fe-, and Mg-containing titanate nanocomposites starting from ilmenite and NaOH and adsorption kinetics, isotherms, and thermodynamics of Cu(II), Cd(II), and Pb(II) cations. Mater. Sci. Eng., B.

[cit46] Kołodyńska D., Krukowska J. A., Thomas P. (2017). Comparison of sorption and desorption studies of heavy metal ions from biochar and commercial active carbon. Chem. Eng. J..

