# Correction: Green synthesis of gum Arabic-activated magnetite–nickel nanoparticles for selective removal of Cd(ii) and Cu(ii) from complex aqueous systems

**DOI:** 10.1039/d5ra90114f

**Published:** 2025-10-10

**Authors:** Entsar H. Taha, Adel A. El-Zahhar, Majed M. Alghamdi, Ahmed M. Masoud, Mohamed F. Kamel, Mohamed H. Taha

**Affiliations:** a Department of Plant Protection, Faculty of Agriculture, Ain Shams University Egypt tahaentesar214@gmail.com; b Department of Chemistry, Faculty of Science, King Khalid University Abha 9004 Saudi Arabia; c Nuclear Materials Authority P.O. Box 530, El Maddi Cairo Egypt

## Abstract

Correction for ‘Green synthesis of gum Arabic-activated magnetite–nickel nanoparticles for selective removal of Cd(ii) and Cu(ii) from complex aqueous systems’ by Entsar H. Taha *et al.*, *RSC Adv.*, 2025, **15**, 35388–35406, https://doi.org/10.1039/D5RA04152J.

The authors regret that the one of the affiliations (affiliation *a*) was incorrectly shown in the original manuscript. The corrected list of affiliations is as shown here.

The Royal Society of Chemistry apologises for these errors and any consequent inconvenience to authors and readers.

