# Supplementary material for: Green synthesis of gum Arabic-activated magnetite–nickel nanoparticles for selective removal of Cd(ii) and Cu(ii) from complex aqueous systems
Source: RSC Adv. 2025 Sep 25;15(42):35388–406. doi: 10.1039/d5ra04152j (PMC12461692; doi:10.1039/d5ra04152j)
Supplement: RA-015-D5RA04152J-s001 [file RA-015-D5RA04152J-s001.pdf]

Supplementary Information (SI) for RSC Advances.
This journal is © The Royal Society of Chemistry 2025

Green synthesis of gum Arabic-activated magnetite–nickel nanoparticles for selective removal of Cd(II) and Cu(II) from complex aqueous systems

Entsar H. Taha [a]****, Adel A. El-Zahhar*** [b]***, Majed M. Alghamdi*** [b] ***, Ahmed M. Masoud*** [C]***, Mohamed F. Kamel*** [C]***, Mohamed H. Taha*** [C]

[a] Department of Plant Protection, Faculty of Agriculture, Ain Shams University, Egypt

Nuclear Materials Authority, P.O. Box 530, El Maddi, Cairo, Egypt

b Department of Chemistry, Faculty of Science, King Khalid University, Abha 9004, Saudi Arabia

[c] Nuclear Materials Authority, P.O. Box 530, El Maddi, Cairo, Egypt

* Corresponding author: tahaentesar214@gmail.com

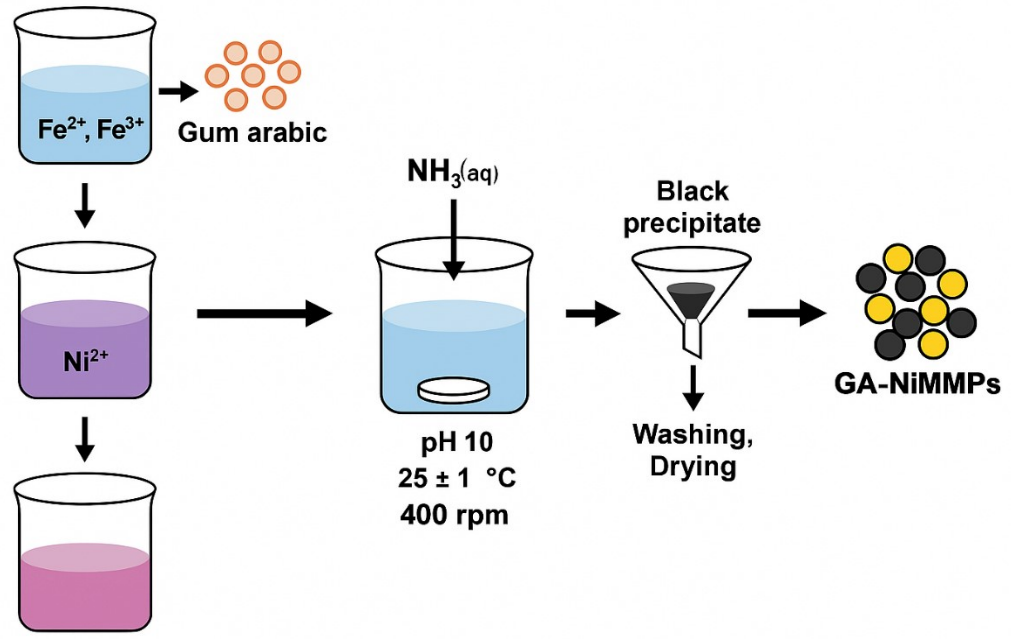

Scheme S1. *Schematic illustration of the synthesis of gum Arabic–activated magnetite–nickel mesoporous nanoparticles (GA-NiMMPs) via green co-precipitation.*

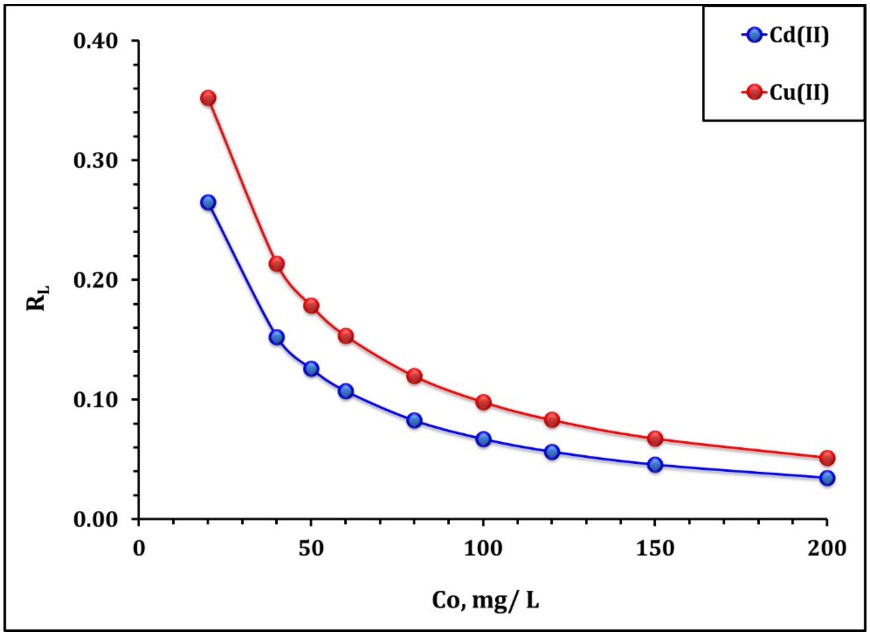

Figure S1: *Separation factor R_L of Cd(II), and Cu(II) adsorption process.*

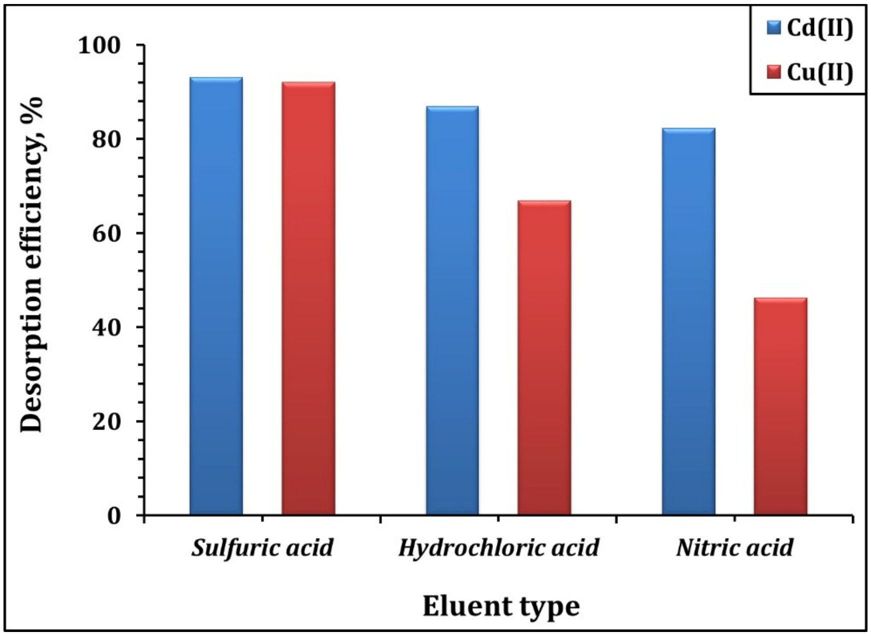

Figure S2: *Cd(II), and Cu(II) desorption from loaded sorbent using different solutions (4.0 g/L, room temperature; 10.0 hrs).*

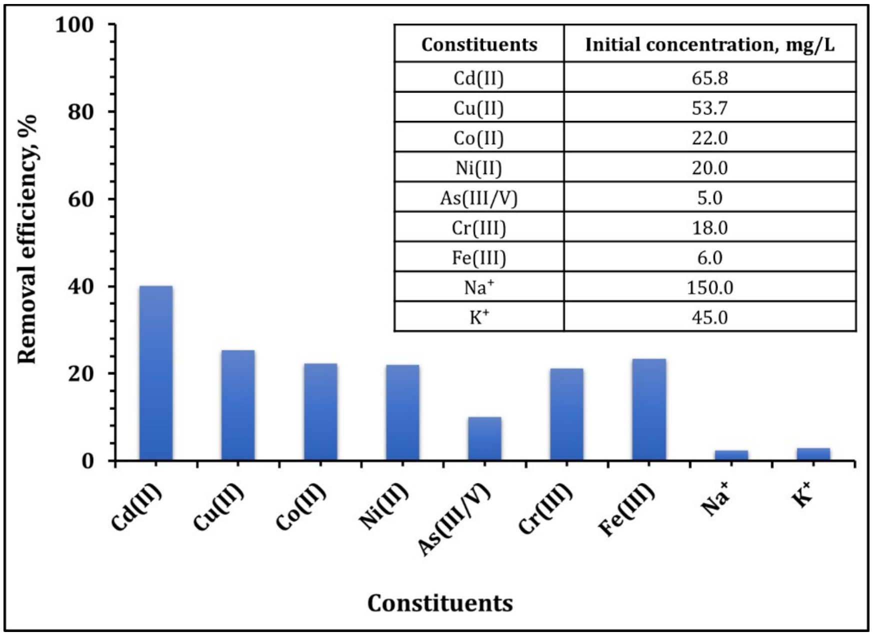

Figure S3: *Initial concentrations of constituents in real raffinate wastewater, along with calculated removal efficiencies after treatment with GA-NiMNPs.*

Table S1: *Kinetic, isotherm, and thermodynamics equations for Cd(II), and Cu(II) sorption process* ***[1-9]***.

**Kinetics**	**Equations**
**Pseudo-first-order**	$q_t = q_1\left(1 - e^{-k1t}\right)$
**Pseudo-second-order**	$q_t = \frac{1}{\left(1 \mid k_2 q_2^2\right) + \left(t \mid q_2\right)}$
**Intra-particle diffusion model (IPD)**	$q_t = K_{id} t^{0.5} + C_i$
**Isotherms**	**Equations**
**Langmuir model**	$q_e = \frac{q_m k_L C_e}{1 + k_L C_e}$
**Freundlich model**	$q_e = K_F C_e^{1/n_F}$
**Temkin model**	$q_e = \frac{RT}{b_T} \ln K_T C_e$
**Sips model**	$q_e = \frac{q_S\left(k_S C_e\right)^{mS}}{1 + \left(k_S C_e\right)^{mS}}$
**Thermodynamics**	**Equations**
$\log K_C = - \frac{\Delta H^o}{2.303\,R} X \frac{1}{T} + C$<br>$-\Delta G^o = 2.303\,RT \log K_C$<br>$\Delta G^o = \Delta H^o - T\,\Delta S^o$	
**Fitting**	**Equations**
**Coordination coefficient (R^2)**	$R^2 = 1 - \frac{\sum_1^n \left(q_{exp} - q_{pred}\right)^2}{\sum_1^n \left(q_{exp} - \bar{q}_{exp}\right)^2}$
**Chi-square coefficient (x^2)**	$x^2 = \sum \left[\frac{\left(q_{exp} - q_{pred}\right)^2}{q_{pred}}\right]$

qe (mg g^{-1}) is the equilibrium concentration of Cd(II) species, and qt (mg g^{-1}) is the adsorbed amount of Cd(II) species ions after time t (min), Ce (mg L^{-1}) is equilibrium concentration of Cd(II) species. k_1 (min^{-1}) and k_2 (min^{-1}) are the rate constants for the pseudo first and second order, respectively. K_{id} (mg/g. $min^{0.5}$) is a rate constant, and C is the thickness of the boundary layer. q_m and q_S are the maximum sorption capacity (mg. g^{-1}) of Langmuir and Sips models. k_L (L. mg^{-1}), K_F (L/ mg), K_T (L min^{-1}), and K_S (L/ mg) are represent the constants of Langmuir, Freundlich, Temkin, and Sips models. n refer to the sorption intensity, b_T is Temkin constant that refers to the adsorption heat, mS is Sips constant. $\boldsymbol{q_s}$ is the theoretical isotherm saturation capacity (mg/g). K_C is a non-dimensional equilibrium constant and it equals K_d X 1000 X ρ **[4-5]**; T is the temperature (K), $\boldsymbol{R}$ is the universal gas constant (8.314 J mol $^{-1}$. K^{-1}), ρ is solution density g/ L, and C is a constant. R^2 and x^2 are the coordination and Chi-square coefficients respectively, the number of test points is n, the experimental equilibrium capacity is q_{exp} (mg g^{-1}), while the predicted capacity is q_{pred} (mg g^{-1}).

References:

1) Hu, Q., Pang, S. and Wang, D., 2022. In-depth insights into mathematical characteristics, selection criteria and common mistakes of adsorption kinetic models: A critical review. *Separation & Purification Reviews*, *51*(3), pp.281-299.
2) González-López, M.E., Laureano-Anzaldo, C.M., Pérez-Fonseca, A.A., Arellano, M. and Robledo-Ortíz, J.R., 2022. A critical overview of adsorption models linearization: methodological and statistical inconsistencies. *Separation & Purification Reviews*, *51*(3), pp.358-372.
3) Shi, L., Deng, Q., Guo, L., Du, Y., Du, D. and Zhang, T.C., 2023. Efficient removal of Cd (II), Cu (II), and Pb (II) in aqueous solutions by exhausted copper slag supported sulfidized nanoscale zerovalent iron. Separation and Purification Technology, 314, p.123483.
4) Niu, H.Y., Li, X. and Li, J., 2022. Dithiocarbamate modification of activated carbon for the efficient removal of Pb (ii), Cd (ii), and Cu (ii) from wastewater. New Journal of Chemistry, 46(11), pp.5234-5245.
5) Chen, X., Hossain, M.F., Duan, C., Lu, J., Tsang, Y.F., Islam, M.S. and Zhou, Y., 2022. Isotherm models for adsorption of heavy metals from water-a review. *Chemosphere*, *307*, p.135545.
6) Majd, M.M., Kordzadeh-Kermani, V., Ghalandari, V., Askari, A. and Sillanpää, M., 2022. Adsorption isotherm models: A comprehensive and systematic review (2010−2020). *Science of The Total Environment*, *812*, p.151334.
7) Taha, M.H., 2021. Sorption of U (VI), Mn (II), Cu (II), Zn (II), and Cd (II) from multi-component phosphoric acid solutions using MARATHON C resin. *Environmental Science and Pollution Research*, *28*(10), pp.12475-12489.
8) Masoud, A.M., Ammar, H., Elzoghby, A.A., El Agamy, H.H. and Taha, M.H., 2024. Rare earth elements adsorption from phosphoric acid solution using dendrimer modified silica gel as well as kinetic, isotherm, and thermodynamic studies. *Journal of Rare Earths*.

9) Ebelegi, A.N., Ayawei, N. and Wankasi, D., 2020. Interpretation of adsorption thermodynamics and kinetics. *Open Journal of Physical Chemistry*, *10*(3), pp.166-182.
